# Adverse events among older adults receiving chiropractic spinal manipulation and related treatments: an updated systematic review

**DOI:** 10.1186/s12998-026-00633-3

**Published:** 2026-03-16

**Authors:** Clinton J. Daniels, Ronald J. Farabaugh, Stacie A. Salsbury, Kristian R. Anderson, Maranda J Kleppe, Wayne M. Whalen, Sheryl A. Walters, Lisa Z. Killinger, Alec L. Schielke

**Affiliations:** 1 VA Puget Sound Health Care System, 9600 Veterans Drive SW, Tacoma, WA USA; 2 University of Washington, Seattle, WA USA; 3 American Chiropractic Association, Highland Park, IL USA; 4 Palmer College of Chiropractic, Davenport, IA USA; 5 Spectra Health, Grand Forks, ND USA; 6 Private Practice, Santee, CA USA; 7 Logan University, Chesterfield, MO USA; 8 Private Practice, Bettendorf, IA USA; 9 VA Palo Alto Health Care System, San Jose, CA USA

**Keywords:** Chiropractic, Spinal manipulation, Geriatrics, Aged, Patient harm, Safety, Adverse effects, Patient safety

## Abstract

**Background:**

This systematic review updated adverse events (AEs) reporting in persons aged > 55 years who received chiropractic treatment, including spinal manipulation.

**Methods:**

Protocol was registered prospectively with the Prospective Register of Systematic Reviews (PROSPERO)(CRD42024629286). Search strategy was audited with Peer Review of Electronic Search Strategies (PRESS) methodology. We searched PubMed, Cochrane Central Register of Controlled Trials, CINAHL, AMED, and Index to Chiropractic Literature on December 12, 2025. We included AEs attributed to treatment provided by chiropractors reported in randomized clinical trials (RCTs), observational studies, and case reports/series. We excluded non-clinical, pilot/feasibility studies, surveys, reviews, populations ≤ 55 years,and papers lacking chiropractic treatment or AE descriptions. Articles were screened and results synthesized by study design and AE characteristics. Study risk of bias (RoB) was assessed in duplicate using Scottish Intercollegiate Guideline Network checklists. Clinical Compass and NCMIC Foundation partially funded this study.

**Results:**

We screened 2295 titles/abstracts, reviewed 125 full-text, and included 25 articles from 6 RCTs, 4 observational studies, and 15 case reports/series. One RCT had low RoB and five acceptable. Three observational cohorts had low RoB and 1 had acceptable. There were 412 study-related adverse events affecting older adult patients, of which 9 were severe, and none were catastrophic. Most adverse events were rated as mild-to-moderate in severity. Reported adverse events consisted of changes in pain quality, muscle or joint soreness or stiffness, numbness, weakness, fatigue, headache, dizziness, or lightheadedness. Several studies lacked AE definitions and/or data collection processes. Case reports described 6 severe AEs; four were vascular in nature. Twenty case reports involved spinal manipulation, one followed TENS application, and one repetitive shoulder abduction, while no case reports described AEs following chiropractor treatment with soft tissue techniques, exercise, or other modalities.

**Conclusion:**

No catastrophic AEs were reported in older adults receiving chiropractic treatment services. Mild-to-moderate symptoms such as muscle soreness and stiffness were common. The lack of standardized AE definitions, severity classifications, and data collection processes across reports limits the certainty. Notable study limitations included a search strategy focused on spinal manipulation, exclusion of administrative database studies that may detect rare AEs, and numerical scoring of RoB checklists.

**Supplementary Information:**

The online version contains supplementary material available at 10.1186/s12998-026-00633-3.

## Background

Patient safety is high priority for chiropractors, especially those treating older adults with musculoskeletal conditions [1–3]. Musculoskeletal pain conditions are common among older adults [4]. Low back and neck pain are the most common conditions seen by chiropractors [5, 6], have the highest prevalence in older adults [7, 8], and age-adjusted disability related to osteoarthritis is on the rise [9, 10]. In addition to age-related changes, high rates of physical, psychosocial, and social factors further complicate the chronic pain presentation for older adults [11]. The odds of spinal pain in older adults increase with subsequent number of comorbidities [12], and spinal pain may precipitate cognitive impairment [13]. Older adults with spinal pain require careful attention during evaluation and diagnosis as serious pathology is not uncommon in this patient group [14, 15].

To manage their musculoskeletal complaints, older adults often use nonpharmacologic therapies provided by chiropractors (e.g., spinal manipulation, exercise, manual therapy modalities) [16]. Spinal manipulation therapy (SMT) is a nonpharmacologic manual therapy approach that is recommended by medical guidelines for both acute and chronic low back pain (LBP) [17, 18]. Older adults who initially use SMT for an episode of chronic LBP have lower rates of care escalation compared to those who use opioid medications as a first treatment [19]. SMT and other manual therapies appear to be well-tolerated and have demonstratable safety profiles in the older adult population [20]. For example, Whedon and associates (2015) reported that older Medicare beneficiaries who received chiropractic treatment had a cumulative probability of injury of 40 incidents per 100,000 patients, which was lower than the 153 injuries per 100,000 patients who had primary care encounters. However, injuries were more common among older chiropractic patients with coagulation problems and those taking anticoagulation treatments, and people with osteoporosis, aortic aneurysm or dissection, and inflammatory spondylopathies [21]. Prevalent comorbidities in older adults, such as frailty [22], fall risk [23], osteoporosis [24, 25], cancer [21], chronic anticoagulant medication use [21, 26], and history of stroke [21, 26] may put patients at higher risk of adverse events (AEs) or be a contraindication to SMT treatment [25].

Adverse events are understood as unfavorable events occurring following interventions, but there is no commonly accepted definition of what qualifies as an AE relative to spinal and peripheral joint manipulation and mobilization [27]. Severity classifications of AEs remain controversial despite efforts to establish consensus terminology [28–30]. Approximately half of chiropractic patients experience AEs (e.g., muscle soreness and stiffness) of mild-to-moderate severity and transient nature following SMT [31]. Further, in the presence of comorbidities, SMT is associated with rare and consequential AEs that may have significant impact on a patient’s well-being, function, and quality of life, such as fracture in the presence of osteoporosis, dislocation in the presence of inflammatory spondylopathy, brain or spinal cord injury when using long-term anticoagulant therapy, and cervical artery dissection [21, 31, 32].

A 2010 systematic review and consensus study, initially updated in 2017, evaluated the safety of chiropractic care for the older adult population and found that a higher rate of AEs were not associated with high-velocity, low-amplitude SMT as compared to low-velocity, low-amplitude SMT or sham [33, 34]. That 2017 update included six papers relevant to adverse events: an RCT [35], an RCT pilot [36], a case series [37], two cohorts of claims data [21, 38], and a narrative review [39]. The prior review did not find any studies on non-SMT treatments offered by chiropractors (e.g., exercise, modalities, advice), and they identified the need to further investigate the safety of managing older adults. Since the prior review, research interest in chiropractic care of the older adult population has led to numerous publications [40, 41]. In addition, the World Federation of Chiropractic has called on the chiropractic profession to actively engage in the advancement of patient safety [42]. Therefore, the purpose of this review was to provide an update on the safety literature on AEs in older adults following chiropractic treatment sessions that included spinal manipulation. Further, the results of this review will inform an updated consensus process on chiropractic management of older adults.

## Methods

### Registration and protocol

The protocol was prospectively registered with the International Prospective Register of Systematic Reviews (PROSPERO) on December 19, 2024 (CRD42024629286) [43]. Study reporting was consistent with the Preferred Reporting of Systematic Reviews and Meta-Analysis (PRISMA) [44].

### Eligibility criteria

The study aim was processed into PICOS (population, intervention, comparison, outcomes, and study design) components to develop eligibility criteria (Table 1).

**Table 1:** Eligibility criteria

Inclusion	Exclusion
Published in peer-reviewed journal February 1st, 2016, through December 12th, 2025 (Start date corresponds to the end date of the most recent systematic review included in the previous clinical practice guideline) Human subjects English language Study population comprised of older adults, 55 years and older, who received treatment services rendered by a chiropractor Studies that evaluate adverse events including: randomized controlled trials, any other clinical trials, and observational studies including case series and case reports	Commentaries/editorials/letters/reviews/ Pilot studies/feasibility studies Non-peer-reviewed publications Surveys and other descriptive cross-sectional studies Conference abstracts Studies that do not address adverse events Study protocols No treatment service outcomes included Non-clinical studies Study population under 55 years of age Treatment service not delivered by a chiropractor

#### Population

The prior systematic review by Hawk et. al defined older adults as 65 and older, however, they included an RCT that enrolled participants aged ≥ 55 years old [mean (SD) age 64.5 (8.9) years] [45]. In this updated systematic review, an a priori decision was made to define older adults as 55 years and over for consistency with Hawk et al. and to maximize the number of eligible studies. This decision was supported by a systematic review of older adults studies inclusion criteria finding a mean age of 61 (SD ± 9.2) years [46] and the BACk Complaints in Elders (BACE) cohort study which included participants ≥ 55 years old [47–49]. We included case reports and clinical studies in which all participants were aged 55 years or older, or observational studies and clinical trials with a mean participant age of approximately 55 years old for which individual data were available.

#### Intervention

Chiropractic treatments were defined as non-pharmacological interventions delivered by a chiropractor. Spinal manipulative therapy was the primary intervention evaluated, but chiropractic treatments also included, but were not limited to, manual therapy, supervised exercise, mind–body interventions, acupuncture, massage therapy, acupressure, electrical modalities, and the application of heat or cold. Treatments could be used independently or in combination.

#### Comparison

No comparison was required.

#### Outcomes

The study or case report was required to provide a description of AEs associated with a chiropractic treatment for inclusion into this review. Adverse events that were deemed by the original study as not-study related were not included. For studies that did not specify if an adverse event was related to the study or external factors, we treated all adverse events reported as study-related. No study level exclusions were made based on whether the authors described the relatedness of AEs to the study.

#### Study design

We included study designs that addressed AEs including randomized controlled trials and other clinical trials, as well as observational designs, such as cohorts, case series and case reports. This deviated from the 2017 review, which had less restrictive inclusion criteria and included pilot studies, claims data, and narrative reviews.

### Information sources

Databases PubMed, Cochrane Central Register of Controlled Trials, CINAHL (Cumulative Index to Nursing and Allied Health Literature), AMED (Allied and Complementary Medicine Database), and ICL (Index to Chiropractic Literature) were searched from the end of the Hawk et al. study through December 18, 2024. The search was last conducted on December 12, 2025. In addition, adverse event articles from the 2017 review by Hawk et al. were included in the screening for eligibility for this updated review [34], and manual citation tracking was performed on included articles to identify potentially relevant articles.

### Search strategy

The search strategy was designed in collaboration with a health sciences librarian (SAW). A second health science librarian assessed the search strategy with the Peer Review of Electronic Search Strategies (PRESS) checklist (Supplemental File A) and additional revisions were made to generate the full search strategy (Supplemental File B). The search terms focused on treatment approaches employed by chiropractors. For example, the search conducted within PubMed, which generated the most returns, included the keywords “adverse effects” paired with the National Library of Medicine Medical Subject Heading (MeSH) for Musculoskeletal Manipulations, which is the preferred (most general) term to identify publications with the following scope: ‘various manipulations of body tissues, muscles and bones by hands or equipment to improve health and circulation, relieve fatigue, promote healing’. MeSH tree structures under this broader heading also were searched, including Manipulation, Chiropractic; Manipulation, Spinal; Manipulation, Osteopathic; and Manipulation, Orthopedic. The search strategy also gathered citations using patient safety terminology (harm, risk, injury, adverse event, adverse effect, etc.) and specific adverse event categories (fracture, cauda equina or complication, artery dissection, etc.).

Chiropractic search terms searched the databases with the wildcard term “chiropract*” (to capture any article with the terms chiropractic/chiropractor/chiropractics/chiropractors) and variations of spinal manipulation. We did not include a comprehensive list of interventions beyond spinal manipulation (e.g., terms related to exercise, soft tissue therapy, acupuncture, dry needling, or passive modalities) as these are not specific to the chiropractic profession. These interventions were included in the analysis when identified through the chiropractic or spinal manipulation related terms.

### Selection process

Citations identified through database search were downloaded into Rayyan, a web application to support screening for systematic reviews [50]. After duplicates were removed, titles and abstracts were independently screened against the eligibility criteria by at least two reviewers (RF, LK, CD). Full text of the potentially eligible articles were independently screened by two reviewers (RF, CD) and disagreements were resolved through discussion. The citations excluded at this stage were tracked with reason recorded.

### Data collection process

Data extraction was completed independently by two groups of paired individuals. Two investigators extracted information from case reports and series (KA, WW), and at least two individuals extracted information from RCTs and cohort studies (MK, SS, CD). Differences were adjudicated through discussion and feedback from a third investigator (CD). Corresponding authors were contacted for eleven studies with a request to provide clarification or for AE data for participants 55 years and older, which was provided by seven authors [51–57], one of which did not meet eligibility criteria [56].

### Data items

Data extracted from eligible RCTs and observational studies included first author surname, publication year, study population, population mean age, sex, condition(s), mean symptom duration, chiropractic treatment group intervention, control group intervention(s), dosage (e.g., number of sessions), AE definition, AE collection method, AEs related to the chiropractic treatment arm(s), and study conclusions. For case reports and series, extraction items included first author surname, publication year, patient age, sex, physical comorbidities, psychological comorbidities, symptoms preceding presentation to the chiropractor, treatment services rendered by the chiropractor, time between treatment and AE occurrence (i.e., 0–2 days, 3–7 days), AE description.

### Study risk of bias assessment

Risk of bias assessment was performed on all included articles. RCTs and observational studies were independently assessed by at least two investigators (MK, SS, CD) using the corresponding study design checklist from the Scottish Intercollegiate Guideline Network (SIGN), consistent with the prior review [58]. The SIGN checklist for RCTs is a 10-question assessment, and the cohort checklist is a 14-question assessment. Case reports and series were independently assessed by two investigators (KA, WW) using the JBI Critical Appraisal Checklist for Case Reports [59]. Disagreements for both teams were resolved through discussion and adjudication by a third investigator (CD). Each checklist question was marked as “1” for “yes” or “0” for “no” or “unclear”, or “NA [not applicable]”. The overall risk of bias assessment for all three tools was determined through the percentage of the total score: greater than or equal to 75% were rated as *high quality, low risk of bias,* between 50 and 75% were rated *moderate quality, acceptable risk of bias,* and 50% or below was deemed *low quality, high risk of bias* and excluded. Not applicable items were deducted from the denominator when calculating percentages.

### Effect measures

We rated the severity of adverse events described in the studies.

### Synthesis methods

We qualitatively synthesized extracted data and organized it by study design, definition of AEs, and AE data collection process. Adverse event severity ratings were classified as 1–4 (1 = mild, 2 = moderate, 3 = severe, 4 = catastrophic [life-threatening]) [30] (Table 2). Body system AEs were operationalized as vascular, non-vascular, or both. We also summarized outcomes by type of intervention, quality of studies, and AEs. For the RCTs and cohort studies, we rated the AEs using the descriptions provided in the studies, and for case reports the classification was determined through discussion of the investigators that independently performed extraction (KA, WW, CD).

**Table 2:** Adverse events severity ratings classification and definitions [30]

Classification (Categorical rating)	Intensity	Definition
Mild (1)	Low intensity, ranging between 1 and 3 on an 11-point numeric scale	No impact on activities, but has a tolerable interference on participation, and quality of life
Moderate (2)	Moderate intensity, ranging between 3 and 6 on an 11-point numeric scale	Some interference with a patient’s activities, participation, and quality of life
Severe (3)	High intensity, ranging between 6 and 8 on an 11-point numeric scale	Not life threatening, but has considerable interference with activities, participation, and quality of life
Catastrophic (4)	Significant intensity; ranging between 8 and 10 on an 11-point numeric scale	Life-threatening and could result in death, totally disrupts activities, participation, and quality of life

### Reporting biases

Meta-analysis and subgroup analysis were not performed. We investigated reporting biases by comparing definitions and collection processes described in the RCT and observational studies.

### Certainty of evidence

Due to the descriptive nature of this systematic review and the wide range of study designs included, we did not assess for certainty.

## Results

### Study selection

**Fig. 1:**
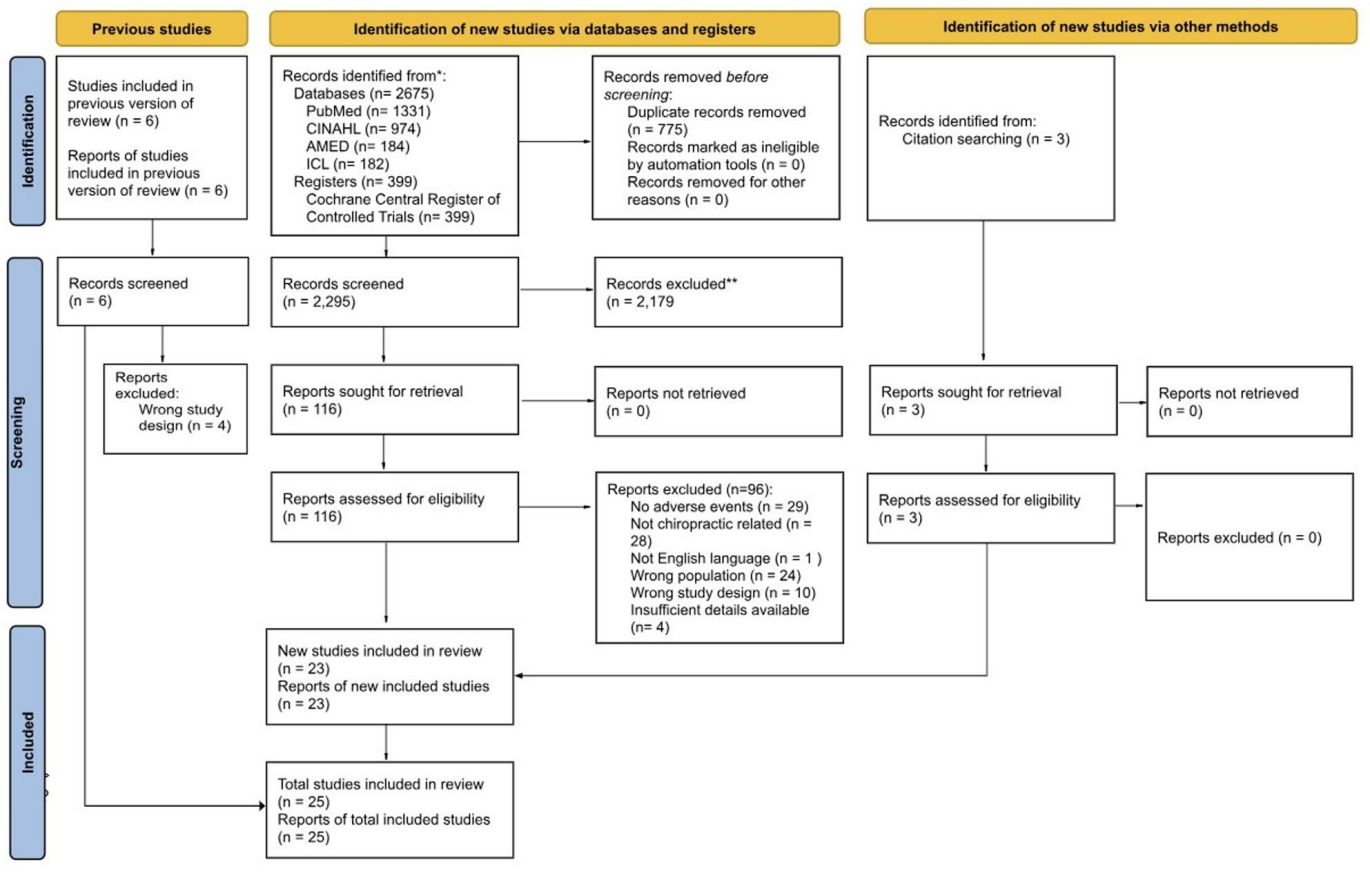
PRISMA flow diagram. Excluded studies listed in Supplementary files

Study investigators screened 2295 titles/abstracts and 125 full-text articles, of which 25 met the eligibility criteria. The selection process is outlined in Fig. 1.

Of the 125 full-text screened articles, 116 were identified from the database and registry search, six were identified from the Hawk et al. 2017 review [34], and an additional three were identified from citation tracking. Ninety-six articles were excluded with reasons provided (Supplemental File C). The most common exclusion reasons were not describing AEs (n = 29), not including an older adult population (n = 24), and describing AEs that did not involve a chiropractor (n = 28). The 25 included articles consisted of six RCTs [35, 53, 54, 60–62], four observational studies [51, 52, 55, 57], and 15 case reports/series [32, 37, 63–75] describing 22 cases.

### Study characteristics

The clinical studies and case series/case reports described 412 adverse events affecting older adult patients related to chiropractic treatment. Most were classified as mild to moderate, eleven were severe, and none were catastrophic. All studies, not exclusively describing severe adverse events, did report mild events (e.g., transient increased pain).

### Study design: RCT

Four RCTs investigated chiropractic treatment of older adults with LBP [35, 53, 60, 62], one studied lumbar spinal stenosis [61], and one low back and neck disability [54]. All six RCTs described study-related interventions that included SMT, as well as chiropractic treatments such as mobilization, flexion-distraction, instrument assisted manipulations, soft tissue massage, exercises or self-care recommendations.

None of the RCTs reported study-related severe or catastrophic AEs. For patients receiving care for LBP, Bronfort et al. described the most common AEs as patients reporting a different type of pain (n = 16), soreness (n = 17), increased pain (n = 8), leg pain, numbness, weakness (n = 8), fatigue (n = 8), and/or dizziness/lightheadedness (n = 8) [53]. Dougherty et al. described 35 AEs definitely/probably associated with the intervention, all of which were mild to moderate soreness, with 42% related to preexisting conditions [35]. Of 414 AEs identified by Goertz et al., 98 were classified as possibly, probably, or definitely related to chiropractic intervention. Of those, seven were rated as moderate severity and 91 mild, with most involving LBP, joint pain, or stiffness [60]. Schulz et al. only collected data on “serious” AEs, and none were determined to be related to the chiropractic treatment arm [62].

For patients with lumbar spinal stenosis, Schneider et al. reported the most common AEs were characterized as muscle soreness (n = 43) and joint soreness (n = 39), with all AEs resolved within 48 h [61]. Maiers et al. reported that six older adult patients receiving care for neck and low back disability experienced a combination of symptoms, including increased neck pain (n = 2), back pain (n = 1), numbness in the hands (n = 2) or feet (n = 2), headache (n = 1), and dizziness with exercise (n = 1) [54].

### Study design: observational

One observational study by Pohlman et al. involved active surveillance of AEs in chiropractic and physical therapy clinics in Canada and the United States [55], a second by Chu et al. reported a retrospective study of all reported AEs following receipt of chiropractic SMT in a Hong Kong medical clinic [52], a third by Gliedt et al. performed a retrospective chart-review investigating for serious adverse events in chiropractic patients with prior spine surgery [57], and a fourth study by Amorin-Woods et al. retrospectively searched 15-years of chiropractic student clinics in Western Australia [51]. Pohlman et al. provided individual patient data and we did not include any adverse events encounter in the physical therapy clinics [55]. Amorin-Woods et al., Chu et al., and Gliedt et al. all provided individual-patient data for all patients 55 years and older [51, 52, 57].

No catastrophic events were reported for the older adult population in any of the observational studies. Out of 54,846 patients, Chu et al. reported 16 older adults with AEs following SMT, most of whom presented for a LBP condition (n = 11), neck pain (n = 3), or an extremity condition (n = 2). Two of the reported AEs were rib fractures, 4 were cases of chest pain without rib fracture, 1 case of jaw pain, and 9 patients with increased pain to the area of chief complaint. Out of 2,136 patients, Pohlman et al. reported 61 older adults experiencing AEs following chiropractic care for LBP (n = 34), neck pain (n = 21), midback pain (n = 17), and/or an extremity condition (n = 26) [55]. None of the AEs were described as serious [catastrophic], five were severe and 18 as moderate, with the most common symptoms being discomfort/pain (n = 22), tiredness/fatigue (n = 9), and numbness (n = 9). Gliedt et al. reported on 174 older adult patients with prior spine surgery, none of which experienced a serious adverse event [57]. Of 61,882 patients, Amorin-Woods et al. reported only 20 AEs and, of those, only 6 involved patients 55 years and older. The AEs experienced by older adults consisted of increased LBP (n = 4), increased neck pain (n = 1), and dizziness and foot numbness (n = 1), all of which they rated as mild [51].

### Study design: case reports

Fifteen case reports described AEs purported as associated with chiropractic treatment. One of the cases described an AE of implantable cardioverter defibrillator (ICD) shock following the application of a transcutaneous electrical nerve stimulation (TENS) unit [71], one dislodged a pacemaker lead with repetitive shoulder abduction [68], and the other 20 were attributed to SMT. Three patients were described as presenting to the chiropractor for LBP, three for a combination of neck, midback, and low back, one for hand numbness, six of chronic obstructive pulmonary disease (COPD), and seven did not describe why the patient presented for chiropractic care. None of the case report AEs were catastrophic, but six were severe; of those, four were vascular [dislodged pacemaker lead [68], epidural hematoma [69], vertebral artery dissection [73], and Page kidney [75]] and two were non-vascular [oral palsies [63] and atlantoaxial dislocation [74]]. Page kidney is a rare condition where compression from an external source triggers secondary hypertension [76]. No case reports described AEs as attributed to soft tissue techniques, exercise, or other physical modalities.

### Risk of bias in studies

One RCT was rated as high quality (low risk of bias), and the other five were rated as acceptable quality (moderate risk of bias) (Table 3). Three of the observational studies were scored as high quality (low risk of bias) and one was rated as acceptable quality (moderate risk of bias) (Table 4). All 15 case reports were described as high-quality by the JBI Critical Appraisal Checklist (Table 5). No studies were excluded for having a high risk of bias.

**Table 3:** Risk of bias assessment of the included randomized controlled trials

SIGN RCTs	Bronfort 2022	Goertz 2017	Maiers 2019	Schneider 2019	Schulz 2019	Dougherty 2014*
1.1	1	1	1	1	1	1
1.2	1	1	1	1	1	1
1.3	1	1	1	1	1	0
1.4	0	0	0	0	0	0
1.5	1	1	1	1	1	1
1.6	0	0	1	0	0	1
1.7	1	1	1	1	1	1
1.8	1	1	1	1	1	1
1.9	1	1	1	1	1	1
1.10	NA	NA	NA	NA	NA	0
Quality	Acceptable	Acceptable	High	Acceptable	Acceptable	Acceptable

* denotes studies also included in the 2017 Hawk et al. review

1.1. The study addresses an appropriate and clearly focused question. 1.2 The assignment of subjects to treatment groups is randomised. 1.3 An adequate concealment method is used. 1.4 The design keeps subjects and investigators ‘blind’ about treatment allocation. 1.5 The treatment and control groups are similar at the start of the trial. 1.6 The only difference between groups is the treatment under investigation. 1.7 All relevant outcomes are measured in a standard, valid and reliable way. 1.8 What percentage of the individuals or clusters recruited into each treatment arm of the study dropped out before the study was completed (≤ 20% = 1, > 20% = 0)? 1.9 All the subjects are analysed in the groups to which they were randomly allocated (often referred to as intention to treat analysis). Where the study is carried out at more than one site, results are comparable for all sites

**Table 4:** Risk of bias assessments for observational cohort studies

	Amorin-Woods 2025	Chu 2023	Gliedt 2025	Pohlman 2024
1.1	1	1	1	1
1.2	NA	NA	NA	NA
1.3	NA	NA	NA	NA
1.4	0	1	1	1
1.5	NA	NA	NA	1
1.6	NA	NA	NA	NA
1.7	1	1	1	1
1.8	NA	NA	NA	1
1.9	1	1	1	1
1.10	1	1	1	1
1.11	0	0	1	1
1.12	NA	NA	NA	1
1.13	1	1	NA	1
1.14	0	1	0	1
Quality	Acceptable	High	High	High

1.1. The study addresses an appropriate and clearly focused question. 1.2 The two groups being studied are selected from source populations that are comparable in all respects other than the factor under investigation. 1.3 The study indicates how many of the people asked to take part did so, in each of the groups being studied. 1.4 The likelihood that some eligible subjects might have the outcome at the time of enrolment is assessed and taken into account in the analysis. 1.5 What percentage of individuals or clusters recruited into each arm of the study dropped out before the study was completed. 1.6 Comparison is made between full participants and those lost to follow up, by exposure status. 1.7 The outcomes are clearly defined. 1.8 The assessment of outcome is made blind to exposure status. If the study is retrospective this may not be applicable. 1.9 Where blinding was not possible, there is some recognition that knowledge of exposure status could have influenced the assessment of outcome. 1.10 The method of assessment of exposure is reliable. 1.11 Evidence from other sources is used to demonstrate that the method of outcome assessment is valid and reliable. 1.12. Exposure level or prognostic factor is assessed more than once. 1.13 The main potential confounders are identified and taken into account in the design and analysis. 1.14 Have confidence intervals been provided?

**Table 5:** Risk of bias assessments for case reports

Case	Baruch 2023	Cohen 2016	Etebari 2023	Garcia 2023	Hall 2018	Ko 2025	Liu 2021	Paulus 2018	Shenoy 2017	Skappak 2018	Szafran 2025	To 2020	Tsou 2019	Wahdat 2017	Dougherty 2011*
1	1	0	1	0	1	1	1	1	1	1	1	1	1	1	1
2	1	1	1	1	1	1	1	1	1	1	1	1	1	1	0
3	1	1	1	1	0	1	1	1	1	1	1	1	1	1	1
4	1	1	1	0	1	1	1	1	1	1	1	1	1	1	1
5	1	1	1	1	0	0	0	1	0	0	1	1	0	0	1
6	1	1	1	1	1	0	1	1	1	1	1	1	1	1	1
7	1	1	1	1	1	1	1	1	1	1	1	1	1	1	1
8	1	1	1	1	1	1	1	0	1	1	1	1	1	1	1
Q	High	High	High	High	High	High	High	High	High	High	High	High	High	High	High

* denotes studies also included in the 2017 Hawk et al. review

*Q* quality

1. Were patient’s demographic characteristics clearly described? 2. Was the patient’s history clearly described and presented as a timeline? 3. Was the current clinical condition of the patient on presentation clearly described? 4 Were diagnostic tests or assessment methods and the results clearly described? 5. Was the intervention(s) or treatment procedure(s) clearly described? 6. Was the post-intervention clinical condition clearly described? 7. Were adverse events (harms) or unanticipated events identified and described? 8. Does the case report provide takeaway lessons?

### Results of individual studies

Data from the extracted RCTs are available in Table 6, observational studies in Table 7, and case reports in Table 8.

**Table 6:** Data extraction of the included randomized controlled trials

Citation and Quality	Population, Mean age (≥ 55 only)	Sex	Condition(s)	Mean symptoms duration	Chiropractic Treatment/ Intervention	Comparison Groups	Dosage	Adverse Event Description as related to chiropractic treatment arms	Conclusion
Bronfort 2022 [53], Acceptable	n = 90, EG: 62.8 (7.3) CG: 64.0 (7.1)	CT: Female n = 26 Male n = 19 IC: Female n = 32 Male n = 11	LBP	CT: 9.7 (12.0) years IC: 7.4 (11.2) years	Chiropractic care alone, may include: HVLA SMT, drop table, mobilization, flexion-distraction, hot and cold packs, exercise, self-care education	Integrative care: chiropractic care plus any combination of acupuncture and oriental medicine, SMT, CBT, exercise, massage, medication, or self-care education	12 weeks intervention period, total # of visits based on patient needs. Mean visits EG 18, CG 24	EG group: diff. type of pain (n = 16); inc. back pain severity (n = 8); new or inc. leg pain, numbness, or weakness (n = 8); unusual or inc. soreness (n = 17); skin irritation (n = 3); more fatigue than normal (n = 8); dizziness/lightheadedness (n = 8); upset stomach, N/V (n = 2); changes in bowel/bladder habits (n = 2) *patients could report more than one AE	Low back pain patients who received integrative care by a multidisciplinary integrative care team tended to have better outcomes than those who received chiropracticcare
Goertz 2017 [61], Acceptable	n = 131,SC: Mean age 73.2 (6.2), Male n = 28, Female = 16; DC: Mean age 72.3 (6.0) MC: Mean age 72.7(6.4)	SC: Female n = 16, Male n = 28; DC: Female n = 16, Male n = 28; MC: Female n = 19, Male n = 24	LBP ≥ 1 month	SC: LBP onset > 1 year 91%; Dual -LBP onset > 1 year 84%; MC: LBP onset > 1 year 77%	DC: (medical + chiro): chiro portion consisted of mobilization, instrument assisted manipulation, SMT, and self care/exercise recommendations SC: (collaborative medical + chiro): same chiro portion above but providers collaborated with each other	MC: self-care and exercise recommendations, medications, and referrals for physical therapy or other health profession	12 weeks	414 AEs, including 6 serious AEs unrelated to intervention and 2 serious AEs unlikely related to intervention Most AEs were judged unrelated (n = 213) or unlikely related (n = 103) to study interventions. Of the 98 AEs classified as possibly (n = 22), probably (n = 31) or definitely (n = 45) related to study interventions, all were rated as mild (n = 91) or moderate (n = 7) in severity. The majority AEs classified as definitely related to study interventions were in the DC and SC groups [MC (n = 2), DC (n = 22), and SC (n = 21)]. Most related AEs (n = 92) involved LBP or joint pain or stiffness attributed to chiropractic care, home exercise or physical therapy, while others (n = 3) included sleep or gastrointestinal complaints	Most adverse events from study interventions included mild musculoskeletal pain or stiffness, which is consistent with AEs reported in other manual therapy trials in older patients
Maiers 2019 [54], High	n = 6, 69.7 (range 65–83)	Female n = 4, Male n = 2	Low back and neck disability ≥ 12 weeks	Unknown	ST: 12 weeks SMT + SEP SMT: HVLA, mobilization, manual distraction, soft tissue massage, stretching SEP: warm-up, stretching, strengthening, balance exercise, self-care education	LT: 36 weeks SMT + SEP SMT: HVLA, mobilization, manual distraction, soft tissue massage, stretching SEP: warm-up, stretching, strengthening, balance exercise, self-care education	SMT: minimum 1 visit/ month to maximum 2 visits/ week SEP: minimum 1 session/ month ST: mean 10 SMT and 4 SEP visits LT: mean 19 SMT and 9 SEP	No serious adverse events were reported. 6 individuals reported mild to moderate adverse events (3/each group) and included 1 or combination of: increase in neck pain (2), back pain (1), numbness in the hands (2) or feet (2), headache (1), and dizziness with exercise (1). Approximately half of participants reported at least 1 side effect over study (51% of total sample at 12 weeks; 58% of those in short-term treatment, 47% in long-term group at 26 weeks). No significant difference between groups was observed in frequency at either time point. An increase or change in neck or back pain was most common	Extending management with SMT and SEP from 12 to 36 weeks did not result in any additional important reduction in disability. Statistically significant differences in favor of long-term management were found for the secondary outcomes of self-reported improvement in neck pain and self-efficacy No SAEs were reported. Cumulatively these findings suggest SMT and SRE are safe for elderly individuals experiencing low back and neck pain related disability
Schneider 2019 [61], Acceptable	n = 259,MC: 72.0 (7.4) GE: 72.9 (8.1) MT/IE: 72.1 (8.1)	MC: Female = 46, Male = 42; GE: Female = 39, Male 45; MT/IE: Female n = 52, Male n = 35	Lumbar spinal stenosis	LBP > 6 months: n = 233 LBP < 6 months: n = 26 LE pain > 6 months: n = 191 LE pain < 6 months: n = 68	MT/IE: (1) Warm-up; (2) spine/hip/ sacroiliac manipulation, distraction mobilization, spine/hip/ neural mobilization; (3) individualized exercise	MC: prescription of oral medications and ability to refer for epidural steroid injections GE: 45 min easy to medium intensity exercise class	Manual therapy/individualized exercise: 2x/wk for 6 wks, 45 min sessions Medical care: 3 visits Group exercise: 2 classes/wk for 6 wks, 45 min classes	MT/IE: soreness (n = 43), joint soreness (n = 39), gastrointestinal complaints (n = 1), headache (n = 1) No SAEs, all AEs resolved within 48 h	MT/IE intervention had better short-term outcomes at 2 months but that none of the interventions were superior to each other at 6 months. All groups showed clinically important improvement in their walking distance, which was sustained at 6 months All adverse events were anticipated minor adverse effects that resolved within 48 h. No serious unanticipated adverse events were found in any group
Schulz 2019 [62], Acceptable	n = 241, SMT + HEP: 72.5 (5.6) SEP + HEP: 73.6 (5.3) HEP: 74.7 (5.6)	SMT + HEP: Female n = 46 Male n = 35 SEP + HEP: Female n = 38 Male n = 42 HEP: Female n = 40 Male n = 40	LBP ≥ 6 weeks Clinical presentation of LBP meeting Quebec Task Force categories of 1, 2, 3, or 4,	SMT + HEP: 13.7 (15.7) years SEP + HEP: 12.1 (15.1) HEP: 12.9 (15.8)	SMT + HEP SMT: HVLA SMT, other manipulation/ mobilization techniques, adjunct therapies HEP: pain self-care, stretching, exercises	SEP + HEP: Similar to HEP with additional exercise and aerobic warm-up HEP: pain self-care, stretching, exercises	SMT + HEP: SMT up to 20 sessions, max 2 per week SEP + HEP: 20 sessions, max 1 per week HEP: 4 sessions, max 1 per week	4 SAEs occurred, all determined to be unrelated to study interventions: 1 hospitalization due to acute cardiac symptoms, 1 new diagnosis prostate cancer, 1 injury attending hockey game, 1 transient ischemic attached during follow-up phase (not actively in intervention)	Adding SMT or SEP to HEP alone does not appear to improve pain or disability outcomes in either the short- or long-term in older adults with chronic LBP, but did enhance satisfaction with care
Dougherty 2014 [35], Acceptable*	n = 136, CT: 76.99 (6.77) Sham: 77.04(6.81)	EG: Female n = 1 Male n = 68 Sham: Female n = 1 Male n = 66	Chronic, mechanical, nonspecific LBP ≥ 3 months	Unknown	HVLA SMT and/or flexion-distraction and/or mobilization, Arthritis Foundation brochure that contained stretching and strengthening exercises	Sham “detuned ultrasound” over lumbar spine for 11 min. Arthritis Foundation brochure that contained stretching and strengthening exercises	Treatment was performed 2 times per week for 4 weeks	**S**MT (n = 141), 35 definitely/probably associated with intervention Most AEs were mild to moderate and were related to MSK soreness; Preexisting conditions accounted for 42% of the AEs, and new events accounted for 58% of the events 6 serious AEs (5 in the SMT group and 1 in sham group). Syncope (n = 2), chest pain (n = 1), paresthesia in the group which was thought to be a myocardial infarction (n = 1), myocardial infarction (n = 1), and fall and injury to neck (n = 1). None of the SAEs were associated with the study interventions	SMT did not demonstrate superiority over sham intervention in older veterans with CLBP in pain. SMT resulted in statistically greater improvement in CLBP-related disability than a sham intervention group at 12-week follow-up, but failed to reach the threshold of clinically significant improvement SMT in older veterans who were naive to chiropractic is safe

* denotes studies also included in the 2017 Hawk et al. review

*AE* adverse event, *CT* chiropractic treatment, *DC* dual care (medical + chiropractic), *GE* group exercise, *HEP* home exercise program, *HVLA SMT* high-velocity, low amplitude spinal manipulative therapy, *IC* integrative care, *LBP* low back pain, *LT* long term, *MC* medical care, *MSK* musculoskeletal, *MT/IE* manual therapy/individualized exercise, *N/V* nausea/vomiting, *SAE* serious adverse events, *SC* shared care (collaborative medical + chiropractic), *SEP* supervised exercise program; ST, short term

**Table 7:** Data extraction of included observational cohorts studies

Citation and Quality	Mean age (≥ 55 only)	Sex	Condition(s)	Mean symptoms duration	Chiropractic Treatment/ Intervention	Comparison Groups	Dosage	Adverse Event Description	Conclusion
Amorin-Woods 2025 [51], Acceptable	n = 6, unavailable	Male n = 4 Female n = 2	Not available for population greater than 55 years old	Not reported	SMT and/or active and passive therapies; Specifically Grade 5 mobilizations (SMT), Soft tissue therapies, Grade 1–4 mobilizations, drop piece, blocking, examination, flexion distraction, stretching, and application of heat	Not Applicable	# of visits: mean = 45, median = 37 (SD 2.12), mode 10, range 10–114	Increased low back pain (n = 4), dizziness and foot numbness (n = 1), increased neck pain (n = 1) All reported AEs were graded 1, mild according to the CTCAE criteria. "All 20 patients experienced either mild or short term signs and/or symptoms; required only clinical or diagnostic observation or treatment modification, and further ongoing intervention, such as medical management, was not reported as required or received."	There were no moderate or severe AEs over 15 years in Western Australia student chiropractic clinics Grade 1 (mild AEs) were rare, yielding a crude (unadjusted) incidence rate of 4.87/100,000 (95% CI: 2.79–7.52) clinical encounters
Chu 2023 [52], High	n = 16,68.2 (10.3)	Male n = 5 Female n = 11	LBP with radiation (n = 8); neck pain (n = 3); axial LBP pain (n = 3); hip pain (n = 1); shoulder pain (n = 1)	Not Reported	SMT (n = 13); SMT + traction (n = 2); SMT + exercise (n = 1)	Not Applicable	# of visits: mean = 27.8, median = 10 # visit AE occurred on: mean = 8.8, median = 5	Increased pain related to chief complaint (n = 9); chest pain without fracture (n = 4); rib fracture (n = 2); jaw pain (n = 1)	No conclusion specific to older adults AE data Low severe AEs consistent with previous studies. The incidence of mild AEs in the present study is much too low to be considered accurate due to data collection methods
Gliedt 2025 [57], High	N = 174, 61.8 (5.6)	Male n = 92, Female n = 82	History of non-cancer and non-scoliosis spine surgery including spinal fusion, cervical disc replacement, microdiscectomy, discectomy with laminectomy/foraminotomy—any of these could be single or multi-level, plus coccygectomy, thoracolumbar spinal cord stimulator implant	Not Reported	Spinal manipulative therapy identified via SMT-specific CPT codes (specific modalities identified in notes: HVLA, table-assisted drop, flexion-distraction, and unknown SMT)	Not Applicable	# of visits mean = 4.3	Post SMT Serious Adverse Event within 10 days of SMT vertebrobasilar/cervical artery injury-VBAI, n = 0; Cauda Equina Syndrome, n = 0; Fracture in SMT region, n = 0; surgical hardware failure, n = 0	There were no incidents of any of the patients receiving SMT experiencing vertebrobasilar/ cervical artery injury, cauda equina syndrome, fracture, or surgical hardware failure within 10 days following SMT
Pohlman 2024 [55], High	n = 61, 65.7 (8.0)	Male n = 21 Female n = 40	Preventative/Wellness/No Symptoms (n = 14) Headache (n = 8) Neck pain (n = 21) Mid-back pain (n = 17) LBP (n = 34) Shoulder/Arm /Knee/Leg Pain (n = 26) Other (n = 9) **more than one could have been marked by each patient	Chronic (n = 39); Acute (n = 16); Missing/ NA (n = 6)	Community-based chiropractors delivering care to consecutive, unique patients. Patients could be either new or returning. SMT was not required to be a given treatment modality	Not Applicable	One visit per patient, as this study assessed AEs after each visit to a unique patient	87 new or worsening symptoms were reported in total mild (n = 64); moderate (n = 18); severe (n = 5); serious (n = 0) discomfort/pain (n = 22); stiffness (n = 13); weakness (n = 4); tiredness/fatigue (n = 9); headache (n = 8); dizziness (n = 4); numbness (n = 9); difficulty walking (n = 7); problems sleeping (n = 7); vomiting/nausea (n = 3); other (n = 1)	No stated conclusion for separate older adults AE data. However, no serious AEs occurred within the older adult population

*AE* adverse event, *LBP* low back pain, *NA* not applicable, *SMT* spinal manipulative therapy

**Table 8:** Data extraction of included case reports

Citation and Quality	Age, Sex	Physical Comorbidities	Psychological Comorbidities	Preceding Symptoms	Chiropractic service(s) rendered	Time between treatment and adverse event onset	Adverse Event Description	Adverse Event Severity Rating	Adverse Event Body System
Baruch 2023 [63], High	59, Male	30–40 pack years smoking	high work stress	Intermittent nocturnal numbness to the right hand	Chiropractic manipulation, bilateral cervical spine	0–2 days	Dyspnea, saliva accumulation, dysphagia, and shortness of breath; dysarthria and hoarseness. ER evaluation concluded injury to CN IX, X, XI, and XII post chiropractic treatment	3	Non-Vascular
Cohen 2016 [64], High	75 Female	none reported	none reported	Neck and back pain	Activator treatment to the suboccipital area	0–2 days	Headache-based acute illness with speech and visual complaints	1	Vascular
Etebari 2023 [65], High	74, Male	bullous pemphygoid on chronic steroids	none reported	Not reported	Drop table adjustment to lumbar and pelvic regions	0–2 days	U-type sacral fracture with forward flexion and anterolisthesis of the S1 segment	2	Non-Vascular
Garcia 2023 [66], High	91, Male	none reported	none reported	Right side low back pain	Activator treatment to the SIJ, gluteal area, and paraspinal region above the iliac crest bilaterally	0–2 days	10/10 pain originating from the left gluteal area with radiation all the way down his left leg to the foot	2	Non-Vascular
Hall 2018 [67], High	61, Male	FXTAS, obesity, hypertension, and obstructive sleep apnea	anxiety, depression	Not reported	"realignment" of neck and back (non-descript)	0–2 days	Deterioration of gait and balance with multiple episodes of vomiting. Acute infarct in the left MCP, atherosclerotic narrowing of the V4 segment of the left vertebral artery, inadequately controlled hypertension, and a LDL of 127	No description of treatment or case outcome	Vascular
Ko 2025 [68], High	83, Male	Pacemaker for AV block at 77 years	None reported	Dyspnea and syncope	Repetitive shoulder abduction to 180 degrees	3–7 days “Several days”	Cardiac perforation and pacing malfunction caused by a dislodged pacemaker lead	3	Vascular
Liu 2021 [69], High	55, Male	None	None reported	Not Reported	Spinal Manipulation	0–2 days	Sudden back pain accompanied by numbness and weakness in both lower limbs, an inability to stand or walk, and difficulty urinating. Reduced sensations, tendon reflexes, and cremaster reflexes of the lower extremities bilaterally	3	Vascular
Paulus 2018 [70], High	59 Female	Headaches, psoriasis, and restless leg syndrome	None reported	Not reported	cervical spinal manipulation, HVLA	0–2 days	Multiple unilateral preretinal hemorrhages with 3 present inferiorly along with a hemorrhage over the optic nerve and a shallow, incomplete posterior vitreous detachment	1	Vascular
Shenoy 2017 [71], High	63 Female	Past medical history significant for hypertension, atrial fibrillation s/p ablation, hypertrophic cardiomyopathy s/p ICD placement, heart failure with an ejection fraction of 40%, left atrial appendage thrombus	None reported	Not reported	Electrical muscle stimulation or TENS (non-specific description)	0–2 days	ICD shock	1	Non-Vascular
Skappak 2018 [72], High	66, Male	Hx of TIA and hypothyroidism; transient pancytopenia two years prior	None reported	low back pain	Chiropractic manipulation (non-descript)	0–2 days	Intensifying low back pain symptoms. Chiropractor recommended further workup after 3 sessions of increased low back pain. Pancytopenia and subacute compression fractures involving the T11, L1, L2, and L3 vertebrae identified; dx with multiple myeloma	2	Non-Vascular
Szafran 2025 [73], High	70, Female	Hypertension and hyperlipidemia	None reported	Subacute neck discomfort	chiropractic neck adjustment, HVLA	0–2 days	Sudden expressive aphasia	3	Vascular
To 2020 [32], High	77, Female	osteopenia, hypertension, hyperlipidemia, hypothyroidism	None reported	Non-specific spinal pains in cervical, thoracic, and lumbar spines	Spinal manipulative therapy	0–2 days	Immediate pain on left side	1	Non-Vascular
	60, Female	osteopenia, smoker, hypertension	Depression	Non-specific spinal pains in cervical, thoracic, and lumbar spines	Spinal manipulative therapy	0–2 days	Immediate pain on her left side over the ribs around the axillary region	1	Non-Vascular
	57, Male	None reported	None reported	Non-specific low back pain	Spinal manipulative therapy	0–2 days	Immediate sharp pain over his left ribs and pain with breathing	1	Non-Vascular
Tsou 2019 [74], High	83, Male	hypertension and old left cerebellar infarction	None reported	Not reported	Chiropractic manipulation (non-descript)	0–2 days	Acute onset left limb weakness. Dislocation of the atlantoaxial joint causing upper cervical spinal cord compression	3	Non-Vascular
Wahdat 2017 [75], High	68, Male	Diabetes mellitus, hypertension, hyperlipidemia, and osteoarthritis	None reported	Not reported	Chiropractic manipulation (non-descript)	0–2 days	Abrupt onset of right flank and groin pain with nausea and nonbloody vomiting; diagnosed Page Kidney	3	Vascular
Dougherty 2011 [37]*	68, Female	Not ambulatory	None reported	Severe COPD	12 sessions in 4 weeks; 2 high-velocity, low-amplitude thoracic manual manipulation, and 1 instrument assisted thoracic manipulation per session	Not reported	No major or moderate AEs were reported after any of the SMT sessions in this study. Minor AEs were reported after 21 of the 72 SMT sessions, with 1 minor AE being reported by each patient. All of these minor AEs resolved within 48 h	1	Non-vascular
	68, Female	Not ambulatory	None reported	Moderate COPD		Not reported		1	Non-vascular
	89, Female	Not ambulatory	None reported	Moderate COPD		Not reported		1	Non-vascular
	86, Female	None reported	None reported	Mild COPD		Not reported		1	Non-vascular
	87, Female	Not ambulatory	None reported	Moderate COPD		Not reported		1	Non-vascular
	77, Male	None reported	None reported	Mild COPD		Not reported		1	Non-vascular

* denotes studies also included in the 2017 Hawk et al. review

*AE* adverse event, *CN* cranial nerve, *COPD* chronic obstructive pulmonary disease, *FXTAS* fragile X-associated tremor/ataxia syndrome, *HVLA SMT* high-velocity, low amplitude spinal manipulative therapy, *Hx* history, *ICD* implantable cardioverter defibrillator, *LBP* low back pain, *MCA* middle cerebral artery, *SIJ* sacroiliac joint, *s/p* status post, *TIA* transient ischemic attack

### Reporting biases

#### Adverse event definitions

Four studies defined AEs as any new or worsening undesirable, untoward, or unfavorable effect [51, 52, 54, 55], two studies defined AEs as any undesirable medical event with new or significant exacerbation [35, 60], and three studies did not provide a definition [53, 61, 62] (Table 9). Pohlman et al. indicated the AE needed to be temporally associated intervention [55], and Dougherty et al. included events up to 30 days following study participation [35]. Gliedt et al. investigated specific serious adverse events, including vertebral artery dissection, cauda equina syndrome, fracture at site of SMT, and hardware failure at site of SMT [57]. Five studies included an adverse event grading scale. Goertz et al., Maiers et al., and Pohlman et al. the events as mild, moderate, severe, or serious [54, 55, 60], Dougherty et al. graded as mild, moderate, or severe [35], and Amorin-Woods et al. used a 1–5 grading scale consistent with mild, moderate, severe, life-threatening, and death [51]. Serious events were defined by these studies as occurrences resulting in death, hospitalization, significant disability, or incapacitation [60, 61]. The classification of AEs as ‘serious’ in these studies was consistent with our use of the ‘catastrophic’ grading category [30].

**Table 9:** Study adverse event definitions and collection methods utilized by the included randomized controlled trial and observational cohort studies

Citation	Study design	Adverse events definition	Adverse events collection method
Bronfort 2022 [53]	RCT	Unknown	Standardized forms
Goertz 2017 [60]	RCT	Defined as any untoward medical occurrence, with serious AEs (SAEs) being those resulting in death, hospitalization, or significant disability or incapacitation. AEs were graded as mild, moderate, severe or serious in severity; expected or unexpected; and definitely, probably, possibly, unlikely or unrelated to any study intervention	Active surveillance process at each study visit
Maiers 2019 [54]	RCT	Anything outside of a side effect (mild increases in pain, muscle soreness, headache, dizziness, or a new MSK pain. All side effects were a) anticipated, b) mild, c) transient, or d) unrelated to treatment.) was classified as an AE Adverse events were categorized by investigators according to standards defined by the U.S. Department of Health and Human Services. (p5)	Patient self-report questionnaire and asked by clinician at each follow-up [visit Active surveillance: Patient self-report questionnaires asked, “since you started treatment in the study have you experienced any of the following?”, followed by a list of side effects known to be associated
Schneider 2019 [61]	RCT	Adverse events was not defined, AEs were characterized as serious (fatal or life-threatening; requires hospitalization or produces a disability) or moderate or greater severity (requires medical evaluation and/or medical treatment; or is a serious adverse reaction) Adverse events were further characterized as unexpected or associated with research intervention	Tracked rates of adverse events via computerized forms. The occurrence of adverse events is monitored for each subject on an ongoing basis throughout the study
Schulz 2019 [62]	RCT	Serious AEs were recorded and classified according to relationship with treatment—exact methodology unknown Additionally, serious adverse events were recorded and classified according to relationship with treatment	Self-reported questionnaires not described in additional detail
Dougherty 2014 [35], Acceptable	RCT	AE was defined as any undesirable medical event with new onset or significant exacerbation during the course of the study (up to 30 adays after conclusion of study participation), regardless of whether or not it was considered to be related to study treatment	Adverse event data were collected at each treatment visit and at the 5- and 12-week follow-up Adverse event data were collected at each treatment visit and at the 5- and 12-week follow-up. Each clinician rated each AE as to severity (a clinical judgment): mild, moderate, or severe. An SAE was defined as any AE occurring during the study or within 30 days of conclusion of study participation, resulting in any one of the following outcomes: death, life-threatening persistent or significant disability/incapacity, hospitalization (when the result of an AE occurring during the study; hospitalization for an elective procedure or for treatment of a preexisting condition not worsened during the study was not considered an SAE; admission to the emergency department for 23 h or less was not considered a hospitalization), congenital anomaly, important medical event
Amorin-Woods 2025 [51]	Cohort	An unexpected or unintended response to treatment/clinical care. ("Excluded from our analysis were those incidents recorded in the register which were not related to clinical care, such as administration errors, equipment failure, public liability type injury and nonclinical communication such as data breaches and failure to improve as expected")	Data regarding AEs used in this study were obtained from an incident register which constituted the records of all patient complaints and clinical incidents between 2008 and 2023
Chu 2023 [52]	Cohort	Any new complaint which is not present at baseline, or a worsening of a presenting complaint compared to baseline	AEs were extracted from a complaint log maintained by a centralized customer service department, with original data sources including solicited patient surveys and phone calls, and non-solicited patient complaints (e.g., emails, phone calls) and clinician reports. Mild soreness following SMT was generally not pursued as an AE. Data regarding AEs were corroborated by medical records data
Gliedt 2025 [57]	Cohort	Defined as to left without general definition of AE or SAE	Retrospective chart audit. For serious adverse events in the short-term, we extracted the following variables: vertebral artery dissection (≤ 10 days post-SMT), cauda equina syndrome (≤ 10 days post-SMT), fracture in location of SMT (≤ 10 days post-SMT), spine surgery hardware failure in same spinal region of SMT application (≤ 10 days post-SMT). We considered each of these serious adverse events as those that were captured by medical records without evidence of complicating/alternative mechanism of injury. All serious adverse events variables were extracted as dichotomous (yes; no) variables
Pohlman 2024 [55]	Cohort	Any unfavorable sign, symptom or disease temporally associated with the treatment, whether or not caused by the treatment; specifically, any new or pre-existing symptom that is worse after treatment	Information on AEs was collected by using three content-validated questionnaires, two completed by the patient and one completed by the provider. Symptoms assessed were: pain/discomfort, stiffness, weakness, fatigue/tiredness, headache, dizziness, numbness/tingling, nausea/vomiting, difficulty walking, problem sleeping, and “other”

*AE* adverse event, *SAE* serious adverse event

### Adverse event collection methods

Five studies used active surveillance with AEs collected at each session [35, 54, 55, 60, 61]. Active surveillance is an intentional and continuous process where safety information is sought from patients and providers through a prespecified collection process, as opposed to passive surveillance where AEs are more commonly reported voluntarily by providers [77]. In addition, Pohlman et al. used three content-validated questionnaires to assess AEs, with two completed by the patient and one by the provider [55]. Three studies used standardized self-reports forms but did not provide additional information on frequency or initiation process [53, 61, 62]. One study reported AEs through a customer service department complaint log, patient surveys and phone calls, and clinician reports [52], and another reported AEs from an incident register that tracks complaints and clinical incidents [51]. Gliedt et al. performed a retrospective chart review with cases identified through presence of surgical CPT codes and chiropractic CPT codes, with eligible charts searched for serious adverse events (i.e. vertebral artery dissection, cauda equina syndrome, fracture in location of SMT, and spine surgery hardware failure in location of SMT) occurring within 10 days following presenting for a chiropractic treatment [57].

## Discussion

We updated the 2017 systematic review [34] on adverse events following SMT and related chiropractic treatments of older adults and identified 19 new studies and case reports. Similar to the prior review, none of the RCTs or observational studies included in this update reported a catastrophic AE. All of the included studies described mild events (e.g., soreness), and there was limited reporting of moderate or severe events (e.g., rib fracture). The lack of catastrophic AEs following SMT is consistent with studies across ages [57, 78–80], suggesting that chiropractic AEs of mild severity are common, but life-threatening AEs are rare.

Our update included five new RCTs, two new observational studies, and 12 new case reports/series. Four studies from the 2017 review were excluded after not meeting the updated study design inclusion criteria [narrative review (n = 1), pilot study (n = 1), descriptive claims data (n = 2)]. None of those studies reported severe or catastrophic AEs, and their inclusion likely would not have substantially changed the findings in this report. Only two studies from the prior review met the updated inclusion criteria, accounting for just 41 of the 412 reported AEs we identified in this update, suggesting substantial growth in reporting of patient safety data for older adult patients since 2017.

By randomizing to minimize bias, use of strict protocols for monitoring, and using comparable groups to establish causation, RCTs are critical for identifying harms in clinical practice [81]. However, RCTs are limited in their ability to detect rare adverse events [82]. The included RCTs and observational cohort studies are powered to assess efficacy but were not adequately powered to observe rare AEs (e.g., cervical artery dissection). Case–control and population-based studies are more appropriate to detect rare events [83]. A 2015 analysis of U.S. Medicare Part B administrative data by Whedon et al. reported that the risk of traumatic injury within 7 days following a chiropractic office visit was increased for individuals with a chronic coagulation defect, inflammatory spondylopathy, osteoporosis, aortic aneurysm, and dissection, or long-term use of anticoagulant therapy [21]. However, the risk of traumatic injury was low, and patients were 76% less likely to have an AE following a chiropractic office visit as compared to a primary care encounter [21]. A second 2015 Medicare B study of adults aged 66–99 years by Whedon et al. reported that the incidence of vertebrobasilar stroke following a chiropractic office visit was extremely low and comparable to those who saw a primary care physician [38]. A study of Medicare Advantage health plan members’ administrative data by Koslof et al. corroborated Whedon et al., reporting no significant association between exposure to chiropractic care and vertebrobasilar artery stroke and concluded that SMT was unlikely to be a cause [84]. A 2025 Medicare claims data of 291,604 patients by Whedon et al. found that Medicare Part B beneficiaries with new onset neck pain were associated with lower rates of adverse events if the patient was seen by a chiropractor as compared to primary medical care [85]. Trager et al. utilized propensity-matching to investigate EHR data for fall risk of older adults (> 65 years) following chiropractic visits and found that the SMT cohort had a lower fall risk than the non-SMT cohort but no difference in limb fracture risk [86]. Their analysis speculated that SMT may reduce fall risk through reducing pain and improving sensorimotor function, but advised caution in interpretation due to the observational design [86]. Like our included clinical studies, administrative and EHR population-based studies suggest that the incidence of severe or catastrophic events following chiropractic visits is rare.

Case reports are not appropriate to make statements about association or causation [87]. However, they were included because can serve as an “early warning system” and bring awareness to rare or previously unknown events that are potentially associated with treatment [81]. One of the case reports described an ICD shock following the application of a TENS unit over the thoracic region. The application of electrical stimulation devices over implantable devices (e.g., ICD, pacemaker) is contraindicated, and providers should be very cautious of applying in nearby regions. The other case reports described AEs following SMT, with them most commonly occurring in the cervical and lumbar regions. Low-impact manipulative treatments are generally thought of as safe, however, one AE described in a case report was following drop table manipulation [65] and two were following treatment with an Activator device [64, 66]. Ten of the cases were reported by chiropractors and ten by medical providers. The premise of one of the Activator case reports [64] was challenged and responded to in letters to the editor [88–90].

Our results present a lack of standardization in the collection and reporting of AEs. There is a need for agreed-upon definitions to be utilized in both routine clinical practice and future investigations of manual therapy treatment harms [27]. An absence of standardized definitions and collection processes sets the stage for underreporting or misinterpretation of risk. The quality (risk of bias) of the included RCTs and observational studies did not appear to correspond to the number of AEs reported. Rather, the volume of AEs reported was dependent on the operational definition of AEs and the data collection methods (active versus passive) utilized. For example, Goertz et al. used active surveillance and identified 98 study related AEs in a study of 131 older adult patients [60], whereas, Amorin-Woods et al. used passive surveillance and identified only 20 AEs (6 in older adults) despite a population of 61,882 patients (all ages) [51].

In ambulatory care settings, the use of active surveillance AE reporting is feasible and results in the collection of significantly more AEs than passive surveillance [91, 92]. A call to action from the World Federation of Chiropractic Global Patient Safety Task Force urged the chiropractic profession to “develop transparent patient safety reporting information systems” with “standard vocabularies and common data models” to build a framework around patient safety [42]. Although it was beyond the scope of this study, the burden of increasing rates of disability secondary to back pain warrant studies to assess how chiropractors can best navigate adverse events in the clinic, educational opportunities to reduce events, and standardize reporting [93].

Since 2017, there has been considerable growth in chiropractic patient safety literature for older adults [34]. Only two studies from the prior review, an RCT [35] and a case series [37] met our inclusion criteria. In the present study, we identified an additional 23 articles over a 8-year period, representing an expansion in the literature on chiropractic treatments for older adults and the study of adverse events. Most of the included RCTs focused on lower back pain or related conditions, and more study is needed on chiropractic treatment approaches for older adults with other conditions (e.g., neck pain, thoracic pain, headaches, extremity conditions). Our study question focused solely on AEs following spinal manipulation treatment encounters with chiropractors. While we did not identify any reports of adverse events following soft tissue techniques or exercise, these approaches are frequently utilized by chiropractors in the management of older adults [6], and AEs for these approaches may be underreported or, in the case of multimodal care, misattributed to spinal manipulation. Future studies should be considered to investigate encounters with other manual therapy providers (e.g., physical therapists, massage therapists, osteopaths). Our study findings bolster the established safety profile for chiropractic treatments and may be utilized to reassure and guide policy makers, payors, and stakeholders in coverage determinations.

## Limitations

There are several limitations to this systematic review. First, and most significantly, our search strategy focused on manipulation-related terminology, which means that most of the articles which were returned in our search focused on SMT. We opted not to search for specific terms for complementary therapies, physical therapy modalities, or rehabilitation interventions that are offered by both chiropractors and other manual therapy providers, such as dry needling, exercise therapy, TENS, or soft tissue therapy. This decision, while focusing our project on a manageable number of citations for screening, also meant that we may have missed important studies or case reports, although the use of the chiropract* wildcard likely captured articles otherwise not included. We encourage researchers planning future systematic reviews of chiropractic interventions to consider broadening their search terms to include these other treatments offered by chiropractors, and to plan their teams, methods, technology, timelines, and budgets to address the higher number of articles that will be returned. We also encourage researchers and clinicians to consider using more robust data collection systems to capture adverse events in their studies and practices for non-SMT treatments.

Secondly, we made two deviations from the protocol. Specifically, we included a study labeled as a pilot despite pilot studies being listed as an exclusion in the eligibility criteria. The study by Goertz et al. was initially excluded at the title/abstract screening phase but was then reconsidered following the urging of a study investigator (SS) [60]. An a priori decision was made to exclude pilot studies because they are generally focused on trial feasibility and underpowered. Despite the Goertz article being titled as a pilot study, it was a large trial that was adequately powered, relevant to our research question, had incorporated an active surveillance process and AE grading classification system, and met all other eligibility criteria. Thus, a team decision was made to include it. It is possible that other pilot studies were excluded at the title/abstract screening phase that may have been relevant to our study. Second, we deviated from our protocol by adding two additional data items: AE operational definitions and approach to collecting AEs. The author team felt including this information provided needed context to frame the AE results of the clinical trials.

Next, we contacted the authors of eleven studies requesting individual patient data. For four of those studies, the authors either could not or would not share their data. It is unclear if their data included any adverse events in patients 55 years and older, or if the inclusion of the data would have impacted our study findings.

Another limitation is that we did not include non-clinical data such as administrative claims data, EHR data, or other databases (e.g., TriNetX). We did not include these sources because they are aggregate data sources that cannot provide granular patient data regarding case-level AEs. However, these study approaches are valuable in recognizing rare AEs, and we provided discussion as relevant to the safety of older adults.

When assessing study risk of bias, we numerically scored each item checklists item and used a percentage to determine overall quality. Scoring without weighting the items has the potential to oversimplify, mask critical flaws and provide misleading summaries of the study [94]. Next, there is no consensus of definitions or terminology for classifying the severity of AEs. We decided a priori to use the terms mild, moderate, severe, and catastrophic based on recent conference proceedings and now published article reporting the results of an e-Delphi study [30, 95]. However, many of the included studies opted for the term *serious* over *catastrophic*, and we treated these terms interchangeably. We assigned severity of AE based on the rating provided in the studies and it is possible the severity was underreported by participating providers. Relatedness of the AE to the intervention also was based on the authors’ judgment, which might differ from the opinions of other adjudicators. That is, some AEs that were related to a chiropractic intervention might not have been labeled as such in the original paper and, therefore, would not have been included in our review. Lastly, we used the SIGN Cohort Checklist to assess the risk of bias in our observational studies. This checklist was designed with a focus on comparing cohorts and is not well suited to retrospective chart reviews [52] or single-arm cohort studies [55]. Therefore, several items from the SIGN cohort checklist were not applicable to the cohorts that met inclusion criteria, and this may have impacted their risk of bias ratings.

## Conclusion

Adverse events following chiropractic treatment services for older adults are common, with examples including changes in pain quality, muscle or joint soreness or stiffness, numbness, weakness, fatigue, headache, dizziness, or lightheadedness. No RCTs or cohort studies involving chiropractic treatment reported study-related catastrophic adverse events in older adults, and all studies reported mild or moderate AEs. The certainty of our findings is limited due to wide variability in the definition and collection process for AEs. Active surveillance of AEs in trials may lead to the identification of more AEs than are currently reported.

## Supplementary Information

Below is the link to the electronic supplementary material.

Supplementary Material 1.

Supplementary Material 2.

Supplementary Material 3.

Supplementary Material 4.

## Data Availability

The datasets will be made available through any reasonable request of the corresponding author. Individual patient data shared by authors of included studies will not be made available and interested parties should contact the corresponding authors of those studies accordingly.
