# Supplementary material for: Adverse events among older adults receiving chiropractic spinal manipulation and related treatments: an updated systematic review
Source: Chiropr Man Therap. 2026 Mar 16;34:15. doi: 10.1186/s12998-026-00633-3 (PMC13104207; doi:10.1186/s12998-026-00633-3)
Supplement: Supplementary file 1 — Supplementary Material 1. [file 12998_2026_633_MOESM1_ESM.docx]

Literature Search for Safety and adverse events associated with chiropractic treatment services among older adults

1166 PubMed

337 Cochrane Central Register of Controlled Trials

837 CINAHL

184 AMED

161 ICL

2685 total results (before deduplication)

PubMed 12.18.24

| #1 | "Manipulation, Chiropractic/adverse effects"[Mesh] or "Manipulation, Spinal/adverse effects"[Mesh] or "Musculoskeletal Manipulations/adverse effects"[Mesh] or "Manipulation, Osteopathic/adverse effects"[Mesh] or "Manipulation, Orthopedic/adverse effects"[Mesh] | 1,290 |
| --- | --- | --- |
| #2 | spinal manipulation*[tiab] or chiropract*[tiab] or “manual therapy”[tiab:~5] or “manual therapies”[tiab:~5] or musculoskeletal manipulation*[tiab] or osteopathic manipulation*[tiab] or orthopedic manipulation*[tiab] or “manipulative therapy”[tiab:~5] or “manipulative therapies”[tiab:~5] or spinal adjustment*[tiab] | 13,739 |
| #3 | patient safety[mh] or harm[tiab] or risk[tiab] or injury[tiab] or "adverse event"[tiab:~5] or "adverse effect"[tiab:~5] or "adverse events"[tiab:~5] or "adverse effects"[tiab:~5] or “artery dissection”[tiab:~5] or fracture*[tiab] or cauda equina or complication*[tiab] or contraindic*[tiab] or death[tiab] or stroke[tiab] or “intra cranial hypotension”[tiab:~5] or “dural tear”[tiab:~5] or paralysis[tiab] or “spinal epidural hematoma”[tiab:~5] or “facet dislocation”[tiab:~5] or “facet subluxation”[tiab:~5] or "para paresis"[tiab:~5] or "hemi paresis"[tiab:~5] or "brown sequard syndrome"[tiab:~5] or "spinal cord injury"[tiab:~5] or "cerebrovascular accident"[tiab:~5] or aneurysm[tiab] | 6,240,875 |
| #4 | #2 and #3 | 3,092 |
| #5 | #1 or #4 | 4,031 |
| #6 | #5 and 2016/02/01:2024[dp] | 1,721 |
| #7 | #6 and English[la] | 1,685 |
| #8 | #7 not (Systematic Review[pt] or meta-analysis[pt] or Review[pt] or Practice Guideline[pt] or Guideline[pt] or systematic[sb]) | 1,166 |

PubMed as search string:

(((("Manipulation, Chiropractic/adverse effects"[Mesh] or "Manipulation, Spinal/adverse effects"[Mesh] or "Musculoskeletal Manipulations/adverse effects"[Mesh] or "Manipulation, Osteopathic/adverse effects"[Mesh] or "Manipulation, Orthopedic/adverse effects"[Mesh]) OR ((spinal manipulation*[tiab] or chiropract*[tiab] or "manual therapy"[tiab:~5] or "manual therapies"[tiab:~5] or musculoskeletal manipulation*[tiab] or osteopathic manipulation*[tiab] or orthopedic manipulation*[tiab] or "manipulative therapy"[tiab:~5] or "manipulative therapies"[tiab:~5] or spinal adjustment*[tiab]) AND (patient safety[mh] or harm[tiab] or risk[tiab] or injury[tiab] or "adverse event"[tiab:~5] or "adverse effect"[tiab:~5] or "adverse events"[tiab:~5] or "adverse effects"[tiab:~5] or "artery dissection"[tiab:~5] or fracture*[tiab] or cauda equina or complication*[tiab] or contraindic*[tiab] or death[tiab] or stroke[tiab] or "intra cranial hypotension"[tiab:~5] or "dural tear"[tiab:~5] or paralysis[tiab] or "spinal epidural hematoma"[tiab:~5] or "facet dislocation"[tiab:~5] or "facet subluxation"[tiab:~5] or "para paresis"[tiab:~5] or "hemi paresis"[tiab:~5] or "brown sequard syndrome"[tiab:~5] or "spinal cord injury"[tiab:~5] or "cerebrovascular accident"[tiab:~5] or aneurysm[tiab]))) and 2016/02/01:2024[dp]) and English[la]) not (Systematic Review[pt] or meta-analysis[pt] or Review[pt] or Practice Guideline[pt] or Guideline[pt] or systematic[sb])

Cochrane Central Register of Controlled Trials 12.18.24

| S1 | MH Manipulation, Chiropractic OR MH Manipulation, Spinal OR MH Musculoskeletal Manipulations OR MH Manipulation, Osteopathic OR MH Manipulation, Orthopedic | Search modes - Proximity | 509 |
| --- | --- | --- | --- |
| S2 | spinal manipulation* OR chiropract* OR manual therapy OR manual therapies OR musculoskeletal manipulation* OR osteopathic manipulation* OR orthopedic manipulation* OR manipulative therapy OR manipulative therapies OR spinal adjustment* | Search modes - Proximity | 6,304 |
| S3 | S1 or S2 | Search modes - Proximity | 6,464 |
| S4 | MH patient safety OR harm OR risk OR injury OR "adverse event" OR "adverse events" OR "adverse effect" OR "adverse effects" | Search modes - Proximity | 650,938 |
| S5 | artery dissection OR fracture OR fractures OR cauda equina OR complication OR complications OR contraindic* OR death OR stroke OR intra cranial hypotension OR dural tear OR paralysis | Search modes - Proximity | 422,595 |
| S6 | spinal epidural hematoma OR facet dislocation OR facet subluxation OR para paresis OR hemi paresis OR brown Sequard syndrome OR spinal cord injury OR cerebrovascular accident OR aneurysm | Search modes - Proximity | 31,112 |
| S7 | S4 OR S5 OR S6 | Expanders - Apply equivalent subjects  Search modes - Proximity | 892,600 |
| S8 | S3 AND S7 | Expanders - Apply equivalent subjects  Search modes - Proximity | 1,480 |
| S9 | S8 | Limiters - Publication Date: 20160201-  Search modes - Proximity | 843 |
| S10 | S9 | Limiters - Publication Date: 20160201-; Language: English  Search modes - Proximity | 635 |
| S11 | S10 | Limiters - Publication Date: 20160201-; Peer Reviewed; Language: English  Search modes – Proximity | 337 |

CINAHL 12.18.24

| S1 | (MH "Manual Therapy+/AE") OR (MH "Manipulation, Orthopedic/AE") OR (MH "Manipulation, Chiropractic/AE") OR (MH "Manipulation, Osteopathic/AE") | Search modes - Proximity | 935 |
| --- | --- | --- | --- |
| S2 | spinal manipulation* OR chiropract* OR manual therapy OR manual therapies OR musculoskeletal manipulation* OR osteopathic manipulation* OR orthopedic manipulation* OR manipulative therapy OR manipulative therapies OR spinal adjustment* | Search modes - Proximity | 42,650 |
| S3 | (MH "Patient Safety") | Search modes - Proximity | 86,037 |
| S4 | harm OR risk OR injury OR "adverse event" OR "adverse events" OR "adverse effect" OR "adverse effects" | Search modes - Proximity | 1,899,913 |
| S5 | artery dissection OR fracture OR fractures OR cauda equina OR complication OR complications OR contraindic* OR death OR stroke OR intra cranial hypotension OR dural tear OR paralysis | Search modes - Proximity | 1,186,146 |
| S6 | spinal epidural hematoma OR facet dislocation OR facet subluxation OR para paresis OR hemi paresis OR brown Sequard syndrome OR spinal cord injury OR cerebrovascular accident OR aneurysm | Search modes - Proximity | 55,621 |
| S7 | S3 OR S4 OR S5 OR S6 | Search modes - Proximity | 2,591,728 |
| S8 | S2 AND S7 | Search modes - Proximity | 6,917 |
| S9 | S1 OR S8 | Search modes - Proximity | 7,106 |
| S10 | S9 | Limiters - Publication Date: 20160201-  Search modes - Proximity | 2,158 |
| S11 | S10 | Limiters - Publication Date: 20160201-; Language: English  Search modes - Proximity | 2,130 |
| S12 | S11 | Limiters - Publication Date: 20160201-; Peer Reviewed; Language: English  Search modes - Proximity | 1,451 |
| S13 | S12 | Limiters - Publication Date: 20160201-; Peer Reviewed; Language: English; Publication Type: Case Study, Clinical Trial, Questionnaire/Scale, Randomized Controlled Trial, Research  Search modes - Proximity | 1,123 |
| S14 | S13 NOT PT systematic review NOT PT Meta Analysis NOT PT Practice Guideline NOT PT Review | Limiters - Publication Date: 20160201-; English Language; Peer Reviewed; Publication Type: Case Study, Clinical Trial, Questionnaire/Scale, Randomized Controlled Trial, Research  Search modes - Proximity | 837 |

AMED 12.18.24

| S1 | spinal manipulation* OR chiropract* OR manual therap* OR musculoskeletal manipulation* OR osteopathic manipulation* OR orthopedic manipulation* OR manipulative therap* OR spinal adjustment* | Search modes - Proximity | 13,301 |
| --- | --- | --- | --- |
| S2 | "patient safety" OR harm OR risk OR injury OR "adverse event" OR "adverse events" OR "adverse effect" OR "adverse effects" | Search modes - Proximity | 59,299 |
| S3 | artery dissection OR fracture OR fractures OR cauda equina OR complication OR complications OR contraindic* OR death OR stroke OR intra cranial hypotension OR dural tear OR paralysis | Search modes - Proximity | 41,439 |
| S4 | spinal epidural hematoma OR facet dislocation OR facet subluxation OR para paresis OR hemi paresis OR brown Sequard syndrome OR spinal cord injury OR cerebrovascular accident OR aneurysm | Search modes - Proximity | 7,608 |
| S5 | S2 OR S3 OR S4 | Search modes - Proximity | 88,557 |
| S6 | S1 AND S5 | Search modes - Proximity | 2,239 |
| S7 | S6 | Limiters - Publication Date: 20160201-  Search modes - Proximity | 287 |
| S8 | S7 | Limiters - Publication Date: 20160201-; Language: English  Expanders - Apply equivalent subjects  Search modes - Proximity | 287 |
| S9 | S8 | Limiters - Publication Date: 20160201-; Language: English; Peer Reviewed  Search modes - Proximity | 184 |

ICL 12.18.24

| [S1](https://chiroindex.org/?action=set&setId=11470414) | Subject:\"Manipulation, Cervical / adverse effects\" OR Subject:\"Manipulation, Chiropractic / adverse effects\" OR Subject:\"Manipulation, Orthopedic / adverse effects\" OR Subject:\"Manipulation, Osteopathic / adverse effects\" OR Subject:\"Manipulation, Spinal / adverse effects\" OR Subject:\"Musculoskeletal Manipulations / adverse effects\", Year: from 2016 to any, Peer Review only | 17 |
| --- | --- | --- |
| [S2](https://chiroindex.org/?action=set&setId=11470423) | All Fields:\"spinal manipulation\" OR All Fields:\"spinal manipulations\" OR All Fields:\"manual therapy\" OR All Fields:\"manual therapies\" OR All Fields:\"musculoskeletal manipulations\" OR All Fields:\"orthopedic manipulation\" OR All Fields:\"orthopedic manipulations\" OR All Fields:\"osteopathic manipulation\" OR All Fields:\"osteopathic manipulations\" OR All Fields:\"manipulative therapy\" OR All Fields:\"manipulative therapies\" OR All Fields:\"spinal adjustment\" OR All Fields:\"spinal adjustments\" OR All Fields:\"musculoskeletal manipulation\", Year: from 2016 to any, Peer Review only | 669 |
| [S3](https://chiroindex.org/?action=set&setId=11470425) | All Fields:\"adverse events\" OR All Fields:\"adverse effects\" OR All Fields:\"adverse event\" OR All Fields:\"adverse effect\" OR All Fields:safety OR All Fields:risk OR All Fields:harm OR All Fields:injury OR All Fields:\"patient safety\" OR All Fields:\"artery dissection\" OR All Fields:fracture OR All Fields:fractures OR All Fields:\"cauda equina\" OR All Fields:complication* OR All Fields:contraindic* OR All Fields:death OR All Fields:stroke OR All Fields:\"intra cranial hypotension\" OR All Fields:\"dural tear\", Year: from 2016 to any, Peer Review only | 578 |
| [S4](https://chiroindex.org/?action=set&setId=11470426) | All Fields:paralysis OR All Fields:\"spinal epidural hypotension\" OR All Fields:\"facet dislocation\" OR All Fields:\"para paresis\" OR All Fields:\"hemi paresis\" OR All Fields:\"brown sequard\" OR All Fields:\"spinal cord injury\" OR All Fields:\"cerebrovascular accident\" OR All Fields:aneurysm, Year: from 2016 to any, Peer Review only | 20 |
| [S5](https://chiroindex.org/?action=set&setId=11470427) | S3 OR S4 | 585 |
| [S6](https://chiroindex.org/?action=set&setId=11470428) | S2 AND S5 | 159 |
| [S7](https://chiroindex.org/?action=set&setId=11470429) | S1 OR S6 | 161 |
