# Supplementary material for: Adverse events among older adults receiving chiropractic spinal manipulation and related treatments: an updated systematic review
Source: Chiropr Man Therap. 2026 Mar 16;34:15. doi: 10.1186/s12998-026-00633-3 (PMC13104207; doi:10.1186/s12998-026-00633-3)
Supplement: Supplementary file 2 — Supplementary Material 2. [file 12998_2026_633_MOESM2_ESM.docx]

# Appendix A

***PRESS Guideline* — Search Submission & Peer Review Assessment**

**SEARCH SUBMISSION: THIS SECTION TO BE FILLED IN BY THE SEARCHER**

| Searcher: Sheryl Walters | Email: Sheryl.Walters@Logan.edu |  |
| --- | --- | --- |
| Date submitted: 12/16/24 | Date requested by: 12/20/24 | *[Maximum = 5 working days]* |

**Systematic Review Title:**

Literature Search Strategy for Safety and adverse events associated with chiropractic treatment services among older adults

This search strategy is …

| X | My PRIMARY (core) database strategy — First time submitting a strategy for search question and database |
| --- | --- |
|  | My PRIMARY (core) strategy — Follow-up review NOT the first time submitting a strategy for search question and database. If this is a response to peer review, itemize the changes made to the review suggestions |
|  | SECONDARY search strategy— First time submitting a strategy for search question and database |
|  | SECONDARY search strategy — NOT the first time submitting a strategy for search question and database. If  this is a response to peer review, itemize the changes made to the review suggestions |

**Database**

PubMed, Cochrane Central Register of Controlled Trials, CINAHL (Cumulative Index to Nursing and Allied Health Literature), Allied and complementary MEDicine (AMED), Index to Chiropractic Literature

**Interface**

MEDLINE through PubMed, CCRCT/CINAHL/AMED through EBSCO

**Research Question**

What are the adverse events associated with chiropractic treatment services among older adults?

**PICO Format**

(Outline the PICOs for your question — i.e., Patient, Intervention, Comparison, Outcome, and Study Design — as applicable)

| **P** | Older adults, 55 years and over |
| --- | --- |
| **I** | Non-pharmacological interventions delivered by a chiropractor |
| **C** | none |
| **O** | adverse events following chiropractic treatment services |
| **S** | randomized controlled trials, any other clinical trials, and observational studies including case series and case reports |

**Inclusion Criteria**

- Published in peer-reviewed journal February 1st, 2016, through TBD/Current search date (This start date corresponds to the end date of the most recent systematic review included in the previous clinical practice guideline)

- Human subjects

- English language

- Study population comprised of older adults, 55 years and older, that received treatment services rendered by a chiropractor

- Studies that evaluate adverse events including: randomized controlled trials, any other clinical trials, and observational studies including case series and case reports

**Exclusion Criteria**

- Commentaries/editorials/letters/reviews/pilot studies/feasibility studies

- Non-peer-reviewed publications

- Surveys and other descriptive cross-sectional studies

- Conference abstracts

- Studies that do not address adverse events

- Study protocols

- No treatment outcomes included

- Non-clinical studies

- Study population under 55 years of age

- Treatment services not delivered by a chiropractor

**Was a search filter applied?**

Yes No

Yes –

PubMed – publication date custom range, language

Cochrane Central Register of Controlled Trials – Custom Date range, English language, and peer reviewed

CINAHL –Custom Date range, English language, and peer reviewed, publication type

AMED - Custom Date range, English language, and peer reviewed

ICL – Year, Peer Review, Publication Type of Randomized Controlled Trial

Other notes or comments you feel would be useful for the peer reviewer? ***[optional]***

The age filter was only used in ICL because every case report has an age subject assigned, unlike PubMed, CCRCT, and CINAHL, which don’t have the indexing for every single record.

Please copy and paste your search strategy here, exactly as run, including the number of hits per line. ***[mandatory]***

PubMed 12.10.24

| #1 | "Manipulation, Chiropractic/adverse effects"[Mesh] or "Manipulation, Spinal/adverse effects"[Mesh] or "Musculoskeletal Manipulations/adverse effects"[Mesh] or "Manipulation, Osteopathic/adverse effects"[Mesh] or "Manipulation, Orthopedic/adverse effects"[Mesh] | 1,289 |
| --- | --- | --- |
| #2 | spinal manipulation[tiab] or chiropract*[tiab] or “manual therapy”[tiab:~5] or “manual therapies”[tiab:~5] or musculoskeletal manipulations[tiab] or osteopathic manipulation[tiab] or orthopedic manipulation[tiab] or “manipulative therapy”[tiab:~5] or “manipulative therapies”[tiab:~5] | 13,668 |
| #3 | patient safety[mh] or harm[tiab] or risk[tiab] or injury[tiab] or "adverse event"[tiab:~5] or "adverse effect"[tiab:~5] or "adverse events"[tiab:~5] or "adverse effects"[tiab:~5] or “artery dissection”[tiab:~5] or fracture*[tiab] or cauda equina or complication*[tiab] or contraindic*[tiab] or death[tiab] or stroke[tiab] or “intra cranial hypotension”[tiab:~5] or “dural tear”[tiab:~5] or paralysis[tiab] or “spinal epidural hematoma”[tiab:~5] or “facet dislocation”[tiab:~5] or “facet subluxation”[tiab:~5] or "para paresis"[tiab:~5] or "hemi paresis"[tiab:~5] or "brown sequard syndrome"[tiab:~5] or "spinal cord injury"[tiab:~5] or "cerebrovascular accident"[tiab:~5] or aneurysm[tiab] | 6,231,726 |
| #4 | #2 and #3 | 3,075 |
| #5 | #1 or #4 | 4,016 |
| #6 | #5 and 2016/02/01:2024[dp] | 1,714 |
| #7 | #6 and English[la] | 1,678 |
| #8 | #7 not (Systematic Review[pt] or meta-analysis[pt] or Review[pt] or Practice Guideline[pt] or Guideline[pt] or systematic[sb]) | 1,162 |

Cochrane Central Register of Controlled Trials 12.10.24

| S1 | MH Manipulation, Chiropractic OR MH Manipulation, Spinal OR MH Musculoskeletal Manipulations OR MH Manipulation, Osteopathic OR MH Manipulation, Orthopedic | Search modes - Proximity | 509 |
| --- | --- | --- | --- |
| S2 | spinal manipulation OR chiropract* OR manual therapy OR manual therapies OR musculoskeletal manipulations OR osteopathic manipulation OR orthopedic manipulation OR manipulative therapy OR manipulative therapies | Search modes - Proximity | 6,300 |
| S3 | S1 or S2 | Search modes - Proximity | 6,461 |
| S4 | MH patient safety OR harm OR risk OR injury OR "adverse event" OR "adverse events" OR "adverse effect" OR "adverse effects" | Search modes - Proximity | 650,938 |
| S5 | artery dissection OR fracture OR fractures OR cauda equina OR complication OR complications OR contraindic* OR death OR stroke OR intra cranial hypotension OR dural tear OR paralysis | Search modes - Proximity | 422,595 |
| S6 | spinal epidural hematoma OR facet dislocation OR facet subluxation OR para paresis OR hemi paresis OR brown Sequard syndrome OR spinal cord injury OR cerebrovascular accident OR aneurysm | Search modes - Proximity | 31,112 |
| S7 | S4 OR S5 OR S6 | Expanders - Apply equivalent subjects  Search modes - Proximity | 892,600 |
| S8 | S3 AND S7 | Expanders - Apply equivalent subjects  Search modes - Proximity | 1,480 |
| S9 | S8 | Limiters - Publication Date: 20160201-  Search modes - Proximity | 843 |
| S10 | S9 | Limiters - Publication Date: 20160201-; Language: English  Search modes - Proximity | 635 |
| S11 | S10 | Limiters - Publication Date: 20160201-; Peer Reviewed; Language: English  Search modes – Proximity | 338 |

CINAHL 12.10.24

| S1 | (MH "Manual Therapy+/AE") OR (MH "Manipulation, Orthopedic/AE") OR (MH "Manipulation, Chiropractic/AE") OR (MH "Manipulation, Osteopathic/AE") | Search modes - Proximity | 946 |
| --- | --- | --- | --- |
| S2 | spinal manipulation OR chiropract* OR manual therapy OR manual therapies OR musculoskeletal manipulations OR osteopathic manipulation OR orthopedic manipulation OR manipulative therapy OR manipulative therapies | Search modes - Proximity | 42,900 |
| S3 | (MH "Patient Safety") | Search modes - Proximity | 85,807 |
| S4 | harm OR risk OR injury OR "adverse event" OR "adverse events" OR "adverse effect" OR "adverse effects" | Search modes - Proximity | 1,898,014 |
| S5 | artery dissection OR fracture OR fractures OR cauda equina OR complication OR complications OR contraindic* OR death OR stroke OR intra cranial hypotension OR dural tear OR paralysis | Search modes - Proximity | 1,185,140 |
| S6 | spinal epidural hematoma OR facet dislocation OR facet subluxation OR para paresis OR hemi paresis OR brown Sequard syndrome OR spinal cord injury OR cerebrovascular accident OR aneurysm | Search modes - Proximity | 55,570 |
| S7 | S3 OR S4 OR S5 OR S6 | Search modes - Proximity | 2,582,169 |
| S8 | S2 AND S7 | Search modes - Proximity | 6,270 |
| S9 | S1 OR S8 | Search modes - Proximity | 6,574 |
| S10 | S9 | Limiters - Publication Date: 20160201-  Search modes - Proximity | 2,149 |
| S11 | S10 | Limiters - Publication Date: 20160201-; Language: English  Search modes - Proximity | 2,121 |
| S12 | S11 | Limiters - Publication Date: 20160201-; Peer Reviewed; Language: English  Search modes - Proximity | 1,444 |
| S13 | S12 | Limiters - Publication Date: 20160201-; Peer Reviewed; Language: English; Publication Type: Case Study, Clinical Trial, Questionnaire/Scale, Randomized Controlled Trial, Research  Search modes - Proximity | 1,121 |
| S14 | S13 NOT PT systematic review NOT PT Meta Analysis NOT PT Practice Guideline NOT PT Review | Limiters - Publication Date: 20160201-; English Language; Peer Reviewed; Publication Type: Case Study, Clinical Trial, Questionnaire/Scale, Randomized Controlled Trial, Research  Search modes - Proximity | 836 |

AMED 12.10.24

| S1 | spinal manipulation OR chiropract* OR manual therap* OR musculoskeletal manipulation* OR osteopathic manipulation OR orthopedic manipulation OR manipulative therap* | Search modes - Proximity | 13,300 |
| --- | --- | --- | --- |
| S2 | "patient safety" OR harm OR risk OR injury OR "adverse event" OR "adverse events" OR "adverse effect" OR "adverse effects" | Search modes - Proximity | 59,299 |
| S3 | artery dissection OR fracture OR fractures OR cauda equina OR complication OR complications OR contraindic* OR death OR stroke OR intra cranial hypotension OR dural tear OR paralysis | Search modes - Proximity | 41,439 |
| S4 | spinal epidural hematoma OR facet dislocation OR facet subluxation OR para paresis OR hemi paresis OR brown Sequard syndrome OR spinal cord injury OR cerebrovascular accident OR aneurysm | Search modes - Proximity | 7,608 |
| S5 | S2 OR S3 OR S4 | Search modes - Proximity | 88,557 |
| S6 | S1 AND S5 | Search modes - Proximity | 2,239 |
| S7 | S6 | Limiters - Publication Date: 20160201-  Search modes - Proximity | 287 |
| S8 | S7 | Limiters - Publication Date: 20160201-; Language: English  Expanders - Apply equivalent subjects  Search modes - Proximity | 287 |
| S9 | S8 | Limiters - Publication Date: 20160201-; Language: English; Peer Reviewed  Search modes - Proximity | 188 |

ICL 12.10.24

| [S1](https://chiroindex.org/?action=set&setId=11470414) | Subject:\"Manipulation, Cervical / adverse effects\" OR Subject:\"Manipulation, Chiropractic / adverse effects\" OR Subject:\"Manipulation, Orthopedic / adverse effects\" OR Subject:\"Manipulation, Osteopathic / adverse effects\" OR Subject:\"Manipulation, Spinal / adverse effects\" OR Subject:\"Musculoskeletal Manipulations / adverse effects\", Year: from 2016 to any, Peer Review only | 17 |
| --- | --- | --- |
| [S2](https://chiroindex.org/?action=set&setId=11470423) | All Fields:\"spinal manipulation\" OR All Fields:\"manual therapy\" OR All Fields:\"manual therapies\" OR All Fields:\"musculoskeletal manipulations\" OR All Fields:\"orthopedic manipulation\" OR All Fields:\"osteopathic manipulation\" OR All Fields:\"manipulative therapy\" OR All Fields:\"manipulative therapies\", Year: from 2016 to any, Peer Review only | 641 |
| [S3](https://chiroindex.org/?action=set&setId=11470425) | All Fields:\"adverse events\" OR All Fields:\"adverse effects\" OR All Fields:\"adverse event\" OR All Fields:\"adverse effect\" OR All Fields:safety OR All Fields:risk OR All Fields:harm OR All Fields:injury OR All Fields:\"patient safety\" OR All Fields:\"artery dissection\" OR All Fields:fracture OR All Fields:fractures OR All Fields:\"cauda equina\" OR All Fields:complication* OR All Fields:contraindic* OR All Fields:death OR All Fields:stroke OR All Fields:\"intra cranial hypotension\" OR All Fields:\"dural tear\", Year: from 2016 to any, Peer Review only | 578 |
| [S4](https://chiroindex.org/?action=set&setId=11470426) | All Fields:paralysis OR All Fields:\"spinal epidural hypotension\" OR All Fields:\"facet disclocation\" OR All Fields:\"para paresis\" OR All Fields:\"hemi paresis\" OR All Fields:\"brown sequard\" OR All Fields:\"spinal cord injury\" OR All Fields:\"cerebrovascular accident\" OR All Fields:aneurysm, Year: from 2016 to any, Peer Review only | 20 |
| [S5](https://chiroindex.org/?action=set&setId=11470427) | All Fields:\"adverse events\" OR All Fields:\"adverse effects\" OR All Fields:\"adverse event\" OR All Fields:\"adverse effect\" OR All Fields:safety OR All Fields:risk OR All Fields:harm OR All Fields:injury OR All Fields:\"patient safety\" OR All Fields:\"artery dissection\" OR All Fields:fracture OR All Fields:fractures OR All Fields:\"cauda equina\" OR All Fields:complication* OR All Fields:contraindic* OR All Fields:death OR All Fields:stroke OR All Fields:\"intra cranial hypotension\" OR All Fields:\"dural tear\", Year: from 2016 to any, Peer Review only OR All Fields:paralysis OR All Fields:\"spinal epidural hypotension\" OR All Fields:\"facet disclocation\" OR All Fields:\"para paresis\" OR All Fields:\"hemi paresis\" OR All Fields:\"brown sequard\" OR All Fields:\"spinal cord injury\" OR All Fields:\"cerebrovascular accident\" OR All Fields:aneurysm, Year: from 2016 to any, Peer Review only | 1071 |
| [S6](https://chiroindex.org/?action=set&setId=11470428) | All Fields:\"spinal manipulation\" OR All Fields:\"manual therapy\" OR All Fields:\"manual therapies\" OR All Fields:\"musculoskeletal manipulations\" OR All Fields:\"orthopedic manipulation\" OR All Fields:\"osteopathic manipulation\" OR All Fields:\"manipulative therapy\" OR All Fields:\"manipulative therapies\", Year: from 2016 to any, Peer Review only AND All Fields:\"adverse events\" OR All Fields:\"adverse effects\" OR All Fields:\"adverse event\" OR All Fields:\"adverse effect\" OR All Fields:safety OR All Fields:risk OR All Fields:harm OR All Fields:injury OR All Fields:\"patient safety\" OR All Fields:\"artery dissection\" OR All Fields:fracture OR All Fields:fractures OR All Fields:\"cauda equina\" OR All Fields:complication* OR All Fields:contraindic* OR All Fields:death OR All Fields:stroke OR All Fields:\"intra cranial hypotension\" OR All Fields:\"dural tear\", Year: from 2016 to any, Peer Review only OR All Fields:paralysis OR All Fields:\"spinal epidural hypotension\" OR All Fields:\"facet disclocation\" OR All Fields:\"para paresis\" OR All Fields:\"hemi paresis\" OR All Fields:\"brown sequard\" OR All Fields:\"spinal cord injury\" OR All Fields:\"cerebrovascular accident\" OR All Fields:aneurysm, Year: from 2016 to any, Peer Review only | 641 |
| [S7](https://chiroindex.org/?action=set&setId=11470429) | Subject:\"Manipulation, Cervical / adverse effects\" OR Subject:\"Manipulation, Chiropractic / adverse effects\" OR Subject:\"Manipulation, Orthopedic / adverse effects\" OR Subject:\"Manipulation, Osteopathic / adverse effects\" OR Subject:\"Manipulation, Spinal / adverse effects\" OR Subject:\"Musculoskeletal Manipulations / adverse effects\", Year: from 2016 to any, Peer Review only OR All Fields:\"spinal manipulation\" OR All Fields:\"manual therapy\" OR All Fields:\"manual therapies\" OR All Fields:\"musculoskeletal manipulations\" OR All Fields:\"orthopedic manipulation\" OR All Fields:\"osteopathic manipulation\" OR All Fields:\"manipulative therapy\" OR All Fields:\"manipulative therapies\", Year: from 2016 to any, Peer Review only AND All Fields:\"adverse events\" OR All Fields:\"adverse effects\" OR All Fields:\"adverse event\" OR All Fields:\"adverse effect\" OR All Fields:safety OR All Fields:risk OR All Fields:harm OR All Fields:injury OR All Fields:\"patient safety\" OR All Fields:\"artery dissection\" OR All Fields:fracture OR All Fields:fractures OR All Fields:\"cauda equina\" OR All Fields:complication* OR All Fields:contraindic* OR All Fields:death OR All Fields:stroke OR All Fields:\"intra cranial hypotension\" OR All Fields:\"dural tear\", Year: from 2016 to any, Peer Review only OR All Fields:paralysis OR All Fields:\"spinal epidural hypotension\" OR All Fields:\"facet disclocation\" OR All Fields:\"para paresis\" OR All Fields:\"hemi paresis\" OR All Fields:\"brown sequard\" OR All Fields:\"spinal cord injury\" OR All Fields:\"cerebrovascular accident\" OR All Fields:aneurysm, Year: from 2016 to any, Peer Review only | 643 |
| [S8](https://chiroindex.org/?action=set&setId=11470431) | All Fields:\"older person\" OR All Fields:\"older persons\" OR All Fields:\"older adult\" OR All Fields:\"older adults\" OR All Fields:\"older patient\" OR All Fields:\"older patients\" OR Subject:\"Aged\" OR Subject:\"Aged, 80 and over\" OR Subject:\"Middle Aged\", Year: from 2016 to any, Peer Review only | 177 |
| [S9](https://chiroindex.org/?action=set&setId=11470432) | Subject:\"Manipulation, Cervical / adverse effects\" OR Subject:\"Manipulation, Chiropractic / adverse effects\" OR Subject:\"Manipulation, Orthopedic / adverse effects\" OR Subject:\"Manipulation, Osteopathic / adverse effects\" OR Subject:\"Manipulation, Spinal / adverse effects\" OR Subject:\"Musculoskeletal Manipulations / adverse effects\", Year: from 2016 to any, Peer Review only OR All Fields:\"spinal manipulation\" OR All Fields:\"manual therapy\" OR All Fields:\"manual therapies\" OR All Fields:\"musculoskeletal manipulations\" OR All Fields:\"orthopedic manipulation\" OR All Fields:\"osteopathic manipulation\" OR All Fields:\"manipulative therapy\" OR All Fields:\"manipulative therapies\", Year: from 2016 to any, Peer Review only AND All Fields:\"adverse events\" OR All Fields:\"adverse effects\" OR All Fields:\"adverse event\" OR All Fields:\"adverse effect\" OR All Fields:safety OR All Fields:risk OR All Fields:harm OR All Fields:injury OR All Fields:\"patient safety\" OR All Fields:\"artery dissection\" OR All Fields:fracture OR All Fields:fractures OR All Fields:\"cauda equina\" OR All Fields:complication* OR All Fields:contraindic* OR All Fields:death OR All Fields:stroke OR All Fields:\"intra cranial hypotension\" OR All Fields:\"dural tear\", Year: from 2016 to any, Peer Review only OR All Fields:paralysis OR All Fields:\"spinal epidural hypotension\" OR All Fields:\"facet disclocation\" OR All Fields:\"para paresis\" OR All Fields:\"hemi paresis\" OR All Fields:\"brown sequard\" OR All Fields:\"spinal cord injury\" OR All Fields:\"cerebrovascular accident\" OR All Fields:aneurysm, Year: from 2016 to any, Peer Review only AND All Fields:\"older person\" OR All Fields:\"older persons\" OR All Fields:\"older adult\" OR All Fields:\"older adults\" OR All Fields:\"older patient\" OR All Fields:\"older patients\" OR Subject:\"Aged\" OR Subject:\"Aged, 80 and over\" OR Subject:\"Middle Aged\", Year: from 2016 to any, Peer Review only | 60 |

**PEER REVIEW ASSESSMENT: THIS SECTION TO BE FILLED IN BY THE REVIEWER**

|  | Reviewer: Jennifer Smith | jennifer.smith@palm | @palmer.edu December 18, 2024  Date completed: | | |
| --- | --- | --- | --- | --- | --- |
|  |  |  |  | | |
|  | **1. TRANSLATION** |  |  | | |
| A -­‐No revisions | | X☐ |  |  |  |
| B -­‐ Revision(s) suggested | | ☐ |  |  |  |
| C -­‐ Revision(s) required | | ☐ |  |  |  |

If “B” or “C,” please provide an explanation or example:

**2. BOOLEAN AND PROXIMITY OPERATORS**

| A -­‐No revisions | X☐ |
| --- | --- |
| B -­‐ Revision(s) suggested | ☐ |
| C -­‐ Revision(s) required | ☐ |

If “B” or “C,” please provide an explanation or example:

**3. SUBJECT HEADINGS**

| A -­‐No revisions | X☐ |
| --- | --- |
| B -­‐ Revision(s) suggested | ☐ |
| C -­‐ Revision(s) required | ☐ |

If “B” or “C,” please provide an explanation or example:

**4. TEXT WORD SEARCHING**

| A -­‐No revisions | ☐ |
| --- | --- |
| B -­‐ Revision(s)suggested | X☐ |
| C -­‐ Revision(s) required | ☐ |

If “B” or “C,” please provide an explanation or example:

In the PubMed search, I would add truncation to the following highlighted terms. This increased my results from 13,679 to 13,724.
**spinal manipulation*[tiab] or chiropract*[tiab] or "manual therapy"[tiab:~5] or "manual therapies"[tiab:~5] or musculoskeletal manipulation*[tiab] or osteopathic manipulation*[tiab] or orthopedic manipulation*[tiab] or "manipulative therapy"[tiab:~5] or "manipulative therapies"[tiab:~5]**

Another phrase to consider adding is spinal adjustment*[tiab]. This increased my results to 13,738

**5. SPELLING, SYNTAX, AND LINE NUMBERS**

| A -­‐No revisions | X☐ |
| --- | --- |
| B -­‐ Revision(s)suggested | ☐ |
| C -­‐ Revision(s) required | ☐ |

If “B” or “C,” please provide an explanation or example:

**6. LIMITS AND FILTERS**

| A -­‐No revisions | ☐ |
| --- | --- |
| B -­‐ Revision(s) suggested | X☐ |
| C -­‐ Revision(s) required | ☐ |

If “B” or “C,” please provide an explanation or example:

I noticed systematic reviews, meta-analyses etc. are filtered in the searches, but not listed in the exclusion criteria. Not sure if they were intended to be on the exclusion list?

Also, not sure if it’s worth the effort, but you could use this search filter to keep adults and remove studies with only infant, child, and/or adolescent populations which I found [here](https://hsls.libguides.com/PubMed-search-filters/limiters). It only reduced my results by 53 in PubMed.
NOT ((infant[mesh] OR child[mesh] OR adolescent[mesh]) NOT adult[mesh])

OVERALL EVALUATION (Note: If one or more “revision required” is noted above, the response below must be “revisions required”.)

| A -­‐No revisions | ☐ |
| --- | --- |
| B -­‐ Revision(s) suggested | x☐ |
| C -­‐ Revision(s) required | ☐ |

Additional comments:

I don't have access to AMED and our subscription to CCRCT is through Cochrane Library, not EBSCO, so the search features are slightly different which made it challenging to duplicate the search. I didn't provide any feedback on those two databases on the PRESS document.
