# Supplementary material for: Adverse events among older adults receiving chiropractic spinal manipulation and related treatments: an updated systematic review
Source: Chiropr Man Therap. 2026 Mar 16;34:15. doi: 10.1186/s12998-026-00633-3 (PMC13104207; doi:10.1186/s12998-026-00633-3)
Supplement: Supplementary file 3 — Supplementary Material 3. [file 12998_2026_633_MOESM3_ESM.docx]

Excluded papers and reasons

**No Adverse Events (n= 29)**

Albano L. Innovative application of Cox Flexion Distraction Decompression to the knee: a retrospective case series. J Can Chiropr Assoc. 2017 Aug;61(2):153-161. PMID: 28928498; PMCID: PMC5596966.

Ammendolia C, Côté P, Rampersaud YR, Southerst D, Schneider M, Ahmed A, Bombardier C, Hawker G, Budgell B. Effect of active TENS versus de-tuned TENS on walking capacity in patients with lumbar spinal stenosis: a randomized controlled trial. Chiropr Man Therap. 2019 Jun 19;27:24. doi: 10.1186/s12998-019-0245-z. PMID: 31244992; PMCID: PMC6582553.

Carlisle C, Polley K, Panda C, Barron K, Hamrock M, Dominique A, Metzger B, Le Brun-Blashka S, Komarnytsky S. Alleviation of Pain, PAIN Interference, and Oxidative Stress by a Novel Combination of Hemp Oil, Calamari Oil, and Broccoli: A Randomized, Double-Blind, Placebo-Controlled Trial. Nutrients. 2023 Jun 7;15(12):2654. doi: 10.3390/nu15122654. PMID: 37375558; PMCID: PMC10304948.

Chow NW, Southerst D, Wong JJ, Kopansky-Giles D, Ammendolia C. Clinical Outcomes in Neurogenic Claudication Using a Multimodal Program for Lumbar Spinal Stenosis: A Study of 49 Patients With Prospective Long-term Follow-up. J Manipulative Physiol Ther. 2019 Mar-Apr;42(3):203-209. doi: 10.1016/j.jmpt.2018.11.004. Epub 2019 Apr 25. PMID: 31029469.

Chu EC. Cervical Radiculopathy as a Hidden Cause of Angina: Cervicogenic Angina. J Med Cases. 2022 Nov;13(11):545-550. doi: 10.14740/jmc4025. Epub 2022 Nov 27. PMID: 36506762; PMCID: PMC9728145.

Chu ECP, Shum JSF, Lin AFC. Unusual Cause of Dysphagia in a Patient With Cervical Spondylosis. Clin Med Insights Case Rep. 2019 Dec 29;12:1179547619882707. doi: 10.1177/1179547619882707. PMID: 31908560; PMCID: PMC6937524.

Chu EC, Trager RJ. Prevalence of Serious Pathology Among Adults with Low Back Pain Presenting for Chiropractic Care: A Retrospective Chart Review of Integrated Clinics in Hong Kong. Med Sci Monit. 2022 Sep 27;28:e938042. doi: 10.12659/MSM.938042. PMID: 36164262; PMCID: PMC9526366.

Chu EC, Trager RJ, Chen ATC. Conservative Management of Low Back Pain Related to an Unresectable Aggressive Sacral Hemangioma: A Case Report. Am J Case Rep. 2022 Jun 23;23:e936984. doi: 10.12659/AJCR.936984. PMID: 35733328; PMCID: PMC9238883.

Chu EC, Trager RJ, Tao C, Lee LY. Chiropractic Management of Neck Pain Complicated by Symptomatic Vertebral Artery Stenosis and Dizziness. Am J Case Rep. 2022 Oct 19;23:e937991. doi: 10.12659/AJCR.937991. PMID: 36258651; PMCID: PMC9597265.

Chu EC, Wong AY, Lin AF. Isolated Neck Extensor Myopathy Associated With Cervical Spondylosis: A Case Report and Brief Review. Clin Med Insights Arthritis Musculoskelet Disord. 2020 Dec 2;13:1179544120977844. doi: 10.1177/1179544120977844. PMID: 33312008; PMCID: PMC7716066.

Haas AL. Resolution of Sleep and Anxiety Disorder Following Reduction of Vertebral Subluxations: A Case Study & Review of the Literature. AJSR 2022;101-113.

Haavik H, Niazi IK, Jochumsen M, Sherwin D, Flavel S, Türker KS. Impact of Spinal Manipulation on Cortical Drive to Upper and Lower Limb Muscles. Brain Sci. 2016 Dec 23;7(1):2. doi: 10.3390/brainsci7010002. PMID: 28025542; PMCID: PMC5297291.

Haavik H, Niazi IK, Jochumsen M, Uginčius P, Sebik O, Yılmaz G, Navid MS, Özyurt MG, Türker KS. Chiropractic spinal manipulation alters TMS induced I-wave excitability and shortens the cortical silent period. J Electromyogr Kinesiol. 2018 Oct;42:24-35. doi: 10.1016/j.jelekin.2018.06.010. Epub 2018 Jun 19. PMID: 29936314.

Holt KR, Haavik H, Lee AC, Murphy B, Elley CR. Effectiveness of Chiropractic Care to Improve Sensorimotor Function Associated With Falls Risk in Older People: A Randomized Controlled Trial. J Manipulative Physiol Ther. 2016 May;39(4):267-78. doi: 10.1016/j.jmpt.2016.02.003. Epub 2016 Apr 2. PMID: 27050038.

Holt K, Niazi IK, Amjad I, Kumari N, Rashid U, Duehr J, Navid MS, Shafique M, Haavik H. The Effects of 4 Weeks of Chiropractic Spinal Adjustments on Motor Function in People with Stroke: A Randomized Controlled Trial. Brain Sci. 2021 May 21;11(6):676. doi: 10.3390/brainsci11060676. PMID: 34064209; PMCID: PMC8224305.

Holt K, Niazi IK, Nedergaard RW, Duehr J, Amjad I, Shafique M, Anwar MN, Ndetan H, Turker KS, Haavik H. The effects of a single session of chiropractic care on strength, cortical drive, and spinal excitability in stroke patients. Sci Rep. 2019 Feb 25;9(1):2673. doi: 10.1038/s41598-019-39577-5. PMID: 30804399; PMCID: PMC6389925.

Houle M, O'Shaughnessy J, Tétreau C, Châtillon CÉ, Marchand AA, Descarreaux M. Comparison of walking variations during treadmill walking test between neurogenic and vascular claudication: a crossover study. Chiropr Man Therap. 2021 Jul 15;29(1):24. doi: 10.1186/s12998-021-00382-5. PMID: 34266463; PMCID: PMC8280597.

Lehman J. Late Whiplash Syndrome - Response to Conservative Chiropractic Management: A Case Report. J Intern Acad Neuromusculoskeletal Med 2025;22(1):13-18.

Minicozzi SJ, Russell B. Increased low back pain in performance of a pelvic tilt maneuver: A report of two cases. JACO 2019;16(1):3-17.

Nunn NR, Battiston A, Harrison J. History, risks, and early referral insights from three reported cases of cervical vascular incidence: The importance of valid informed consent for cervical spine adjustments. Asia-Pac Chiropr J 2025;6(2):1-21.

Oakley PA, Jaeger JO, Brown JE, Polatis TA, Clarke JG, Whittler CD, Harrison DE. The CBP^®^ mirror image^®^ approach to reducing thoracic hyperkyphosis: a retrospective case series of 10 patients. J Phys Ther Sci. 2018 Aug;30(8):1039-1045. doi: 10.1589/jpts.30.1039. Epub 2018 Jul 24. PMID: 30154597; PMCID: PMC6110211.

Olson HM, Zetocha AJ, Olson CA. Chiropractic Management of a Patient With Perineal Numbness After Arthroscopic Hip Surgery: A Case Report. J Chiropr Med. 2016 Dec;15(4):305-309. doi: 10.1016/j.jcm.2016.08.002. Epub 2016 Oct 13. PMID: 27857639; PMCID: PMC5106423.

Perrucci RM, Coulis CM. Chiropractic management of post spinal cord stimulator spine pain: a case report. Chiropr Man Therap. 2017 Feb 6;25:5. doi: 10.1186/s12998-017-0136-0. PMID: 28191306; PMCID: PMC5292803.

Pocovi NC, Kent P, Lin CC, French SD, de Campos TF, da Silva T, Hancock MJ. Recurrence of low back pain: A difficult outcome to predict. Development and validation of a multivariable prediction model for recurrence in patients recently recovered from an episode of non-specific low back pain. Musculoskelet Sci Pract. 2023 Apr;64:102746. doi: 10.1016/j.msksp.2023.102746. Epub 2023 Mar 11. PMID: 36948043.

Rodewald C, Steward T, Fridinger S. Non-surgical chiropractic management of a massive lumbar disc extrusion: A case report. J Contemp Chiropr 2025;8(1):68-74.

Russell BS, Hosek RS, Hoiriis KT, Drake ED. Chronic Progressive External Ophthalmoplegia and Bilateral Vestibular Hypofunction: Balance, Gait, and Eye Movement Before and After Multimodal Chiropractic Care: A Case Study. J Chiropr Med. 2019 Jun;18(2):144-154. doi: 10.1016/j.jcm.2018.11.004. Epub 2019 Jul 1. PMID: 31367202; PMCID: PMC6656914.

Wong AYL, Parent EC, Dhillon SS, Prasad N, Samartzis D, Kawchuk GN. Differential patient responses to spinal manipulative therapy and their relation to spinal degeneration and post-treatment changes in disc diffusion. Eur Spine J. 2019 Feb;28(2):259-269. doi: 10.1007/s00586-018-5851-2. Epub 2019 Jan 2. PMID: 30604298.

Young BA, Boland DM, Manzo A, Yaw H, Carlson B, Carrier S, Corcoran K, Dial M, Briggs RB, Tragord B, Koppenhaver SL. Immediate Effects of Adding Dry Needling to Thoracic Manipulation and Exercise in Cervical Range of Motion for Adults With Neck Pain: A Randomized Clinical Trial. J Manipulative Physiol Ther. 2022 Sep;45(7):531-542. doi: 10.1016/j.jmpt.2022.10.002. Epub 2022 Dec 13. PMID: 36517270.

Young JJ, Kopansky-Giles D, Ammendolia C. Multimodal non-surgical intervention for individuals with knee osteoarthritis: a retrospective case series. J Can Chiropr Assoc. 2019 Aug;63(2):92-99. PMID: 31564747; PMCID: PMC6743648.

**No Chiropractor Involved in Treatment (n= 28)**

Abbasi S, Hadian Rasanani MR, Ghotbi N, Olyaei GR, Bozorgmehr A, Rasouli O. Short-term effect of kinesiology taping on pain, functional disability and lumbar proprioception in individuals with nonspecific chronic low back pain: a double-blinded, randomized trial. Chiropr Man Therap. 2020 Nov 20;28(1):63. doi: 10.1186/s12998-020-00349-y. PMID: 33213492; PMCID: PMC7678105.

Alshami AM, Alghamdi MA, Abdelsalam MS. Effect of Neural Mobilization Exercises in Patients With Low Back-Related Leg Pain With Peripheral Nerve Sensitization: A Prospective, Controlled Trial. J Chiropr Med. 2021 Jun;20(2):59-69. doi: 10.1016/j.jcm.2021.07.001. Epub 2021 Oct 2. PMID: 34987322; PMCID: PMC8703155.

Atıcı E., Gül K., Kardes K., Tütüneken Y.E., Dürüstkan Elbaşı N., Buran Cirak Y. Immediate effect of manual therapy on respiratory functions and respiratory muscle strength in stroke patients. Turk J Physiother Rehabil 2024;35(3):335-341.

Baeske R, Hall T, Dall'Olmo RR, Silva MF. In people with shoulder pain, mobilisation with movement and exercise improves function and pain more than sham mobilisation with movement and exercise: a randomised trial. J Physiother. 2024 Oct;70(4):288-293. doi: 10.1016/j.jphys.2024.08.009. Epub 2024 Sep 25. PMID: 39327172.

Boucher J, Mooney S, Dewey T, Kirtley RG, Walker T, Rabago D. Manual Therapy Informed by the Fascial Distortion Model for Plantar Heel Pain: Results of a Single-Arm Prospective Effectiveness Study. J Altern Complement Med. 2021 Aug;27(8):697-705. doi: 10.1089/acm.2020.0486. Epub 2021 Jun 29. PMID: 34185582.

Carlisle C, Polley K, Panda C, Barron K, Hamrock M, Dominique A, Metzger B, Le Brun-Blashka S, Komarnytsky S. Alleviation of Pain, PAIN Interference, and Oxidative Stress by a Novel Combination of Hemp Oil, Calamari Oil, and Broccoli: A Randomized, Double-Blind, Placebo-Controlled Trial. Nutrients. 2023 Jun 7;15(12):2654. doi: 10.3390/nu15122654. PMID: 37375558; PMCID: PMC10304948.

Castaldo M, Catena A, Chiarotto A, Fernández-de-Las-Peñas C, Arendt-Nielsen L. Do Subjects with Whiplash-Associated Disorders Respond Differently in the Short-Term to Manual Therapy and Exercise than Those with Mechanical Neck Pain? Pain Med. 2017 Apr 1;18(4):791-803. doi: 10.1093/pm/pnw266. PMID: 28034987.

Castro-Sánchez AM, Gil-Martínez E, Fernández-Sánchez M, Lara-Palomo IC, Nastasia I, de Los Ángeles Querol-Zaldívar M, Aguilar-Ferrándiz ME. Manipulative therapy of sacral torsion versus myofascial release in patients clinically diagnosed posterior pelvic pain: a consort compliant randomized controlled trial. Spine J. 2021 Nov;21(11):1890-1899. doi: 10.1016/j.spinee.2021.05.002. Epub 2021 May 13. PMID: 33991702.

Cha JY, Choi S, Kim S, Jang IT. Vertebral Body Fracture During Manual Therapy in a Patient Following Spinal Surgery and Previous Instrumentation Removal. Korean J Neurotrauma. 2025 Jul 18;21(3):216-221. doi: 10.13004/kjnt.2025.21.e23. PMID: 40778253; PMCID: PMC12325884.

Chen Y, Mofatteh M, Nguyen TN, Wellington J, Wei W, Liang W, Chen G, Hu Z, Ouyang K, Yang S. Carotid Artery Dissection and Ischemic Stroke Following Cervical Chiropractic Manipulation: Two Case Reports. Vasc Endovascular Surg. 2022 Apr;56(3):303-307. doi: 10.1177/15385744211072660. Epub 2021 Dec 31. PMID: 34971321.

Choi JY, Lee JI. Extracranial vertebral artery rupture likely secondary to "cupping therapy" superimposed on spontaneous dissection. Interv Neuroradiol. 2017 Apr;23(2):156-158. doi: 10.1177/1591019916685081. Epub 2017 Jan 17. PMID: 28304198; PMCID: PMC5433608.

Cho J, Lee E, Lee S. Effectiveness of mid-thoracic spine mobilization versus therapeutic exercise in patients with subacute stroke: A randomized clinical trial. Technol Health Care. 2019;27(2):149-158. doi: 10.3233/THC-181467. PMID: 30664514.

Engel RM, Bailey R, Luker C, Parry A, Graham P, Grace S. The effect of manual therapy and low intensity exercise on lung function and quality of life in healthy adults between 50 and 65 years: a randomised controlled trial. Journal of the Australian Traditional-Medicine Society. 2020;26(3)158-163.

Ferreira CCQ, Lima KR, Dias-Peixoto MF, Orlando DR, Castelo PM, Pereira LJ, Francelino Andrade E. Manual therapy and dry needling improve mobility, pain and reduce fear of COVID-19 in temporomandibular disorder patients: Randomized controlled trial. J Bodyw Mov Ther. 2024 Oct;40:620-626. doi: 10.1016/j.jbmt.2024.05.015. Epub 2024 May 27. PMID: 39593654.

Grannò S, Lavé A, Molliqaj G, Jannelli G, Schaller K, Tessitore E, Nouri A. Sudden spinal cord injury after cervicothoracic manipulation therapy: illustrative case. J Neurosurg Case Lessons. 2025 Aug 11;10(6):CASE25304. doi: 10.3171/CASE25304. PMID: 40789223; PMCID: PMC12337983.

Griffin K, O'Hearn M, Franck CC, Courtney CA. Passive accessory joint mobilization in the multimodal management of chronic dysesthesia following thalamic stroke. Disabil Rehabil. 2019 Aug;41(16):1981-1986. doi: 10.1080/09638288.2018.1450453. Epub 2018 Mar 20. PMID: 29557687.

Haller H, Lauche R, Cramer H, Rampp T, Saha FJ, Ostermann T, Dobos G. Craniosacral Therapy for the Treatment of Chronic Neck Pain: A Randomized Sham-controlled Trial. Clin J Pain. 2016 May;32(5):441-9. doi: 10.1097/AJP.0000000000000290. PMID: 26340656; PMCID: PMC4894825.

Maicki T, Trąbka R, Wilk-Frańczuk M, Krzepkowska W. Proprioceptive neuromuscular facilitation therapy versus manual therapy in patients with neck pain: a randomised controlled trial. J Rehabil Med. 2024 Sep 5;56:jrm40002. doi: 10.2340/jrm.v56.40002. PMID: 39235053; PMCID: PMC11558861.

Murray KJ, Molyneux T, Le Grande MR, Castro Mendez A, Fuss FK, Azari MF. Association of Mild Leg Length Discrepancy and Degenerative Changes in the Hip Joint and Lumbar Spine. J Manipulative Physiol Ther. 2017 Jun;40(5):320-329. doi: 10.1016/j.jmpt.2017.03.001. Epub 2017 Apr 18. PMID: 28427725.

Nambi G, Alghadier M, Khanam H, Pakkir Mohamed SH, Aldhafian OR, Alshahrani NA, Mani P, Chevidikunnan MF, Khan F, Albarakati AJA. An Additive Effect of Instrument-Assisted Soft Tissue Mobilization with Spinal Manipulation in Cervicogenic Headache: a Randomized Controlled Trial. Pain Ther. 2024 Dec;13(6):1679-1693. doi: 10.1007/s40122-024-00671-w. Epub 2024 Oct 28. PMID: 39467979; PMCID: PMC11544115.

Naseri F, Dadgoo M, Pourahmadi M, Amroodi MN, Azizi S, Shamsi A. Dry needling as an adjunct treatment to multimodal rehabilitation protocol following rotator cuff repair surgery: a preliminary, randomized sham-controlled trial. Chiropr Man Therap. 2024 Dec 5;32(1):39. doi: 10.1186/s12998-024-00555-y. PMID: 39639410; PMCID: PMC11622656.

Peters R, Mutsaers B, Verhagen AP, Koes BW, Pool-Goudzwaard AL. Prospective Cohort Study of Patients With Neck Pain in a Manual Therapy Setting: Design and Baseline Measures. J Manipulative Physiol Ther. 2019 Sep;42(7):471-479. doi: 10.1016/j.jmpt.2019.07.001. Epub 2019 Nov 23. PMID: 31771834.

Prall J, Dunning J, Young I, Ross M, Escaloni J, Bliton P. Pain and Disability Reduction Following Rib Manipulation in a Patient Recovering from Osteomyelitis of the Thoracic Spine. Healthcare (Basel). 2025 Jun 6;13(12):1355. doi: 10.3390/healthcare13121355. PMID: 40565382; PMCID: PMC12192616.

Sampsonis T, Karanasios S, Gioftsos G. The Immediate Hypoalgesic Effects of Mobilization and Manipulation in Patients with Non-Specific Chronic Low Back Pain: A Cross-Over Randomized Controlled Trial. Healthcare (Basel). 2025 Jul 17;13(14):1719. doi: 10.3390/healthcare13141719. PMID: 40724744; PMCID: PMC12294813.

Stanek JM, Pieczynski AE. Effectiveness of clinician- and patient-applied mobilization with movement technique to increase ankle dorsiflexion range of motion. Int J Ther Rehab 2020;27(4). Doi: 10.12968/ijtr.2018.0118

Truque-Díaz C, Pérez-Llanes R, Meroño-Gallut J, Cuesta-Barriuso R, Donoso-Úbeda E. Efficacy and Safety of Manual Therapy in Haemophilic Ankle Arthropathy: A Randomised Crossover Clinical Trial. Healthcare (Basel). 2025 Sep 5;13(17):2228. doi: 10.3390/healthcare13172228. PMID: 40941580; PMCID: PMC12428218.

Wang F, Wang X, Li X, Zheng H, Zhang Z. A case of multiple Vertebrobasilar artery fenestration misdiagnosed as vertebral artery dissection. BMC Neurol. 2020 Feb 20;20(1):63. doi: 10.1186/s12883-020-01642-2. PMID: 32079531; PMCID: PMC7033924.

Wetzler G, Roland M, Fryer-Dietz S, Dettmann-Ahern D. CranioSacral Therapy and Visceral Manipulation: A New Treatment Intervention for Concussion Recovery. Med Acupunct. 2017 Aug 1;29(4):239-248. doi: 10.1089/acu.2017.1222. PMID: 28874926; PMCID: PMC5580370.

**Not available in English language (n=1)**

Durán Vian C, Salgüero Fernández I, Alfageme Roldán F, Roustán Gullón G. Subcutaneous Neck Lesion After Chiropractic Manipulation: The Role of Ultrasound Skin Imaging. Actas Dermosifiliogr (Engl Ed). 2020 Mar;111(2):183-185. English, Spanish. doi: 10.1016/j.ad.2018.08.012. Epub 2019 Oct 13. PMID: 31615630.

**Wrong Population (n= 24)**

Alanazi S, Alawfi AM, Alrashedan BS, Almohaini RA, Shogair MM, Alshehri TA. Spinal Accessory Nerve Injury following Spinal Adjustment: Case Report and Literature Review of the Outcome of Accessory Nerve Pathology as Result of Blunt Trauma (Spinal Accessory Nerve Palsy after Spinal Adjustment). Case Rep Orthop. 2024 Feb 29;2024:7440745. doi: 10.1155/2024/7440745. PMID: 38456196; PMCID: PMC10919972.

Bozer JJ, Helfrich KM, Seidler GR, Garrett AL, Hussain N. A Case of a Cerebrospinal Fluid Leak Secondary to Chiropractic Manipulation of the Thoracic Spine. A A Pract. 2024 Sep 9;18(9):e01844. doi: 10.1213/XAA.0000000000001844. PMID: 39248364.

Bush HM, Stanek JM, Wooldridge JD, Stephens SL, Barrack JS. Comparison of the Graston Technique® With Instrument-Assisted Soft Tissue Mobilization for Increasing Dorsiflexion Range of Motion. J Sport Rehabil. 2020 Nov 25;30(4):587-594. doi: 10.1123/jsr.2019-0397. PMID: 33238244.

Chaibi A, Benth JŠ, Tuchin PJ, Russell MB. Chiropractic spinal manipulative therapy for migraine: a three-armed, single-blinded, placebo, randomized controlled trial. Eur J Neurol. 2017 Jan;24(1):143-153. doi: 10.1111/ene.13166. Epub 2016 Oct 2. PMID: 27696633; PMCID: PMC5214068.

Chaibi A, Knackstedt H, Tuchin PJ, Russell MB. Chiropractic spinal manipulative therapy for cervicogenic headache: a single-blinded, placebo, randomized controlled trial. BMC Res Notes. 2017 Jul 24;10(1):310. doi: 10.1186/s13104-017-2651-4. PMID: 28738895; PMCID: PMC5525198.

Chan TLH, Kim DD, Sharma M, Jog M. Hypoglossal Nerve Palsy Following Chiropractic Neck Manipulation. Can J Neurol Sci. 2019 Sep;46(5):633-634. doi: 10.1017/cjn.2019.237. Epub 2019 Jul 31. PMID: 31248469.

Crothers AL, French SD, Hebert JJ, Walker BF. Spinal manipulative therapy, Graston technique® and placebo for non-specific thoracic spine pain: a randomised controlled trial. Chiropr Man Therap. 2016 May 16;24:16. doi: 10.1186/s12998-016-0096-9. Erratum in: Chiropr Man Therap. 2016 Jul 11;24:31. doi: 10.1186/s12998-016-0111-1. PMID: 27186365; PMCID: PMC4868028.

Cunningham J, Hoskins W, Ferris S. Upper Trunk Brachial Plexus Palsy Following Chiropractic Manipulation. Front Neurol. 2016 Nov 30;7:211. doi: 10.3389/fneur.2016.00211. PMID: 27965621; PMCID: PMC5127815.

Eklund A, Jensen I, Lohela-Karlsson M, Hagberg J, Leboeuf-Yde C, Kongsted A, Bodin L, Axén I. The Nordic Maintenance Care program: Effectiveness of chiropractic maintenance care versus symptom-guided treatment for recurrent and persistent low back pain-A pragmatic randomized controlled trial. PLoS One. 2018 Sep 12;13(9):e0203029. doi: 10.1371/journal.pone.0203029. PMID: 30208070; PMCID: PMC6135505.

Gevers-Montoro C, Romero-Santiago B, Medina-García I, Larranaga-Arzamendi B, Álvarez-Gálovich L, Ortega-De Mues A, Piché M. Reduction of Chronic Primary Low Back Pain by Spinal Manipulative Therapy is Accompanied by Decreases in Segmental Mechanical Hyperalgesia and Pain Catastrophizing: A Randomized Placebo-controlled Dual-blind Mixed Experimental Trial. J Pain. 2024 Aug;25(8):104500. doi: 10.1016/j.jpain.2024.02.014. Epub 2024 Feb 16. PMID: 38369221.

Gorrell LM, Beath K, Engel RM. Manual and Instrument Applied Cervical Manipulation for Mechanical Neck Pain: A Randomized Controlled Trial. J Manipulative Physiol Ther. 2016 Jun;39(5):319-329. doi: 10.1016/j.jmpt.2016.03.003. Epub 2016 May 12. PMID: 27180949.

Hartnett DA, Milner JD, Kleinhenz DT, Kuris EO, Daniels AH. Malpractice Litigation Involving Chiropractic Spinal Manipulation. World Neurosurg. 2021 May;149:e108-e115. doi: 10.1016/j.wneu.2021.02.067. Epub 2021 Feb 23. PMID: 33631389.

Kennell KA, Daghfal MM, Patel SG, DeSanto JR, Waterman GS, Bertino RE. Cervical artery dissection related to chiropractic manipulation: One institution's experience. J Fam Pract. 2017 Sep;66(9):556-562. PMID: 28863201.

Kerr MS, Yawman JP, Kelly JJ, Shaath MK, Langford JR. Chiropractic Manipulation Causing Anterior-Posterior Compression Type Pelvic Ring Injury: A Case Report. JBJS Case Connect. 2023 Dec 8;13(4). doi: 10.2106/JBJS.CC.23.00291. PMID: 38064577.

Li W, Chang Y, Feng Q, Cheng Y, Yin J, Sun Y, Yang F. Balance chiropractic therapy for cervical spondylotic radiculopathy: A randomized controlled trial. Contemp Clin Trials Commun. 2024 Jul 14;41:101323. doi: 10.1016/j.conctc.2024.101323. PMID: 39188412; PMCID: PMC11345501.

Mitra A, Azad HA, Prasad N, Shlobin NA, Cloney MB, Hopkins BS, Jahromi BS, Potts MB, Dahdaleh NS. Chiropractic associated vertebral artery dissection: An analysis of 34 patients amongst a cohort of 310. Clin Neurol Neurosurg. 2021 Jul;206:106665. doi: 10.1016/j.clineuro.2021.106665. Epub 2021 Apr 24. PMID: 34020327.

Nam JJ, Koh Y, Park JW, Choi IC. Fascicular Injury in True Neurogenic Thoracic Outlet Syndrome Associated with Manual and Shockwave Therapies: A Case Report. J Brachial Plex Peripher Nerve Inj. 2025 Oct 7;20(1):e83-e86. doi: 10.1055/a-2700-4984. PMID: 41064729; PMCID: PMC12503974.

Plachinski SJ, Gliedt JA, Sacho R, Schneider MJ, King JA. Spinal manipulative therapy and cervical artery dissection: A retrospective comparison with spontaneous, traumatic, and iatrogenic etiologies at a single academic medical center. Clin Neurol Neurosurg. 2021 Oct;209:106941. doi: 10.1016/j.clineuro.2021.106941. Epub 2021 Sep 11. PMID: 34547642.

Ramos GB, Ranzani Martins R, Carvalhinho Carlos de Souza J, Falcão FCSEB, Lopes CCB, Gargioni de Andrade AL, Silva GD. Spinal Cord Injury, Vertebral Artery Dissection, and Cerebellar Strokes After Chiropractic Manipulation. Neurology. 2022 Nov 29;99(22):995-996. doi: 10.1212/WNL.0000000000013078. Epub 2021 Nov 18. Erratum in: Neurology. 2022 Jul 5;99(1):42. doi: 10.1212/WNL.0000000000200334. PMID: 34795047.

Sutton J, Mann M, Zuckerman M. Man With Headache. Ann Emerg Med. 2022 Jan;79(1):e1-e2. doi: 10.1016/j.annemergmed.2021.07.111. PMID: 34949418.

Tabell V, Tarkka IM, Holm LW, Skillgate E. Do adverse events after manual therapy for back and/or neck pain have an impact on the chance to recover? A cohort study. Chiropr Man Therap. 2019 Jun 12;27:27. doi: 10.1186/s12998-019-0248-9. PMID: 31205681; PMCID: PMC6560736.

Tak I, Bertrand B, Laghout R, et al. A manual muscle manipulation and early return to sports in footballers with long-standing adductor-related groin pain; a prospective case series. Msk Sci Pract 2016;25:E108.

Trager RJ, Baumann AN, Perez JA, Dusek JA, Perfecto RT, Goertz CM. Association between chiropractic spinal manipulation and cauda equina syndrome in adults with low back pain: Retrospective cohort study of US academic health centers. PLoS One. 2024 Mar 11;19(3):e0299159. doi: 10.1371/journal.pone.0299159. PMID: 38466710; PMCID: PMC10927125.

Uhrenholt L. Serious bicycle crash injury in chiropractic practice - a case report of delayed diagnosis. Chiropr Man Therap. 2016 Nov 1;24:40. doi: 10.1186/s12998-016-0121-z. PMID: 27822361; PMCID: PMC5088667.

**Wrong Study Design (n= 10)**

Alcantara J, Whetten A, Ohm J, Alcantara J. Ratings of perceived effectiveness, patient satisfaction and adverse events experienced by wellness chiropractic patients in a practice-based research network. Complement Ther Clin Pract. 2019 Aug;36:82-87. doi: 10.1016/j.ctcp.2019.06.003. Epub 2019 Jun 8. PMID: 31383450.

Brown S. Review of nine malpractice cases with allegations of causation of cervical artery dissection by cervical spine manipulation: No evidence for causation. J Forensic Leg Med. 2024 Nov;108:102783. doi: 10.1016/j.jflm.2024.102783. Epub 2024 Oct 22. PMID: 39454519.

Chaibi A, Allen-Unhammer A, Køpke Vøllestad N, Russell MB. Chiropractic spinal manipulative therapy for acute neck pain: A 4-arm clinical placebo randomized controlled trial. A prospective study protocol. PLoS One. 2023 Dec 7;18(12):e0295115. doi: 10.1371/journal.pone.0295115. PMID: 38060549; PMCID: PMC10703251.

Meerwijk EL, Larson MJ, Schmidt EM, Adams RS, Bauer MR, Ritter GA, Buckenmaier C 3rd, Harris AHS. Nonpharmacological Treatment of Army Service Members with Chronic Pain Is Associated with Fewer Adverse Outcomes After Transition to the Veterans Health Administration. J Gen Intern Med. 2020 Mar;35(3):775-783. doi: 10.1007/s11606-019-05450-4. Epub 2019 Oct 28. PMID: 31659663; PMCID: PMC7080907.

Regan KA, Youn TS, Iyer SSR. Ophthalmic artery occlusion after chiropractic neck manipulation. Acta Ophthalmol. 2018 Aug;96(5):e663-e664. doi: 10.1111/aos.13738. Epub 2018 May 31. PMID: 29855170.

Whedon JM, Kizhakkeveettil A, Toler AW, Bezdjian S, Rossi D, Uptmor S, MacKenzie TA, Lurie JD, Hurwitz EL, Coulter I, Haldeman S. Initial Choice of Spinal Manipulation Reduces Escalation of Care for Chronic Low Back Pain Among Older Medicare Beneficiaries. Spine (Phila Pa 1976). 2022 Feb 15;47(4):E142-E148. doi: 10.1097/BRS.0000000000004118. PMID: 34474443; PMCID: PMC8581066.

Whedon JM, Kizhakkeveettil A, Toler AW, MacKenzie TA, Lurie JD, Hurwitz EL, Bezdjian S, Bangash M, Uptmor S, Rossi D, Haldeman S. Initial Choice of Spinal Manipulative Therapy for Treatment of Chronic Low Back Pain Leads to Reduced Long-term Risk of Adverse Drug Events Among Older Medicare Beneficiaries. Spine (Phila Pa 1976). 2021 Dec 15;46(24):1714-1720. doi: 10.1097/BRS.0000000000004078. PMID: 33882542; PMCID: PMC8629350.

Whedon JM, Petersen CL, Li Z, Schoelkopf WJ, Haldeman S, MacKenzie TA, Lurie JD. Association between cervical artery dissection and spinal manipulative therapy -a medicare claims analysis. BMC Geriatr. 2022 Nov 29;22(1):917. doi: 10.1186/s12877-022-03495-5. PMID: 36447166; PMCID: PMC9710172.

Whedon JM, Toler AWJ, Goehl JM, Kazal LA. Association Between Utilization of Chiropractic Services for Treatment of Low Back Pain and Risk of Adverse Drug Events. J Manipulative Physiol Ther. 2018 Jun;41(5):383-388. doi: 10.1016/j.jmpt.2018.01.004. Epub 2018 May 26. PMID: 29843912.

Whedon JM, Uptmor S, Toler AWJ, Bezdjian S, MacKenzie TA, Kazal LA Jr. Association between chiropractic care and use of prescription opioids among older medicare beneficiaries with spinal pain: a retrospective observational study. Chiropr Man Therap. 2022 Jan 31;30(1):5. doi: 10.1186/s12998-022-00415-7. PMID: 35101064; PMCID: PMC8802278.

**Inadequate Details Available (n= 4)**

Chaibi A, Benth JŠ, Tuchin PJ, Russell MB. Adverse events in a chiropractic spinal manipulative therapy single-blinded, placebo, randomized controlled trial for migraineurs. Musculoskelet Sci Pract. 2017 Jun;29:66-71. doi: 10.1016/j.msksp.2017.03.003. Epub 2017 Mar 14. Erratum in: Musculoskelet Sci Pract. 2017 Oct;31:21. doi: 10.1016/j.msksp.2017.06.001. PMID: 28324697.

Chong JD, Langlois NEI, Byard RW. A retrospective search of postmortem examination reports indicates that death following chiropractic neck manipulation in Australia appears to be a rare event. J Forensic Sci. 2025 May;70(3):1089-1093. doi: 10.1111/1556-4029.70007. Epub 2025 Feb 24. PMID: 39992032.

Petrozzi MJ, Leaver A, Ferreira PH, Rubinstein SM, Jones MK, Mackey MG. Addition of MoodGYM to physical treatments for chronic low back pain: A randomized controlled trial. Chiropr Man Therap. 2019 Oct 25;27:54. doi: 10.1186/s12998-019-0277-4. PMID: 31673330; PMCID: PMC6814139.

Suwaidi ASA, Moustafa IM, Kim M, Oakley PA, Harrison DE. A Comparison of Two Forward Head Posture Corrective Approaches in Elderly with Chronic Non-Specific Neck Pain: A Randomized Controlled Study. J Clin Med. 2023 Jan 9;12(2):542. doi: 10.3390/jcm12020542. PMID: 36675471; PMCID: PMC9861410.
